# Modeling UV/Vis Absorption Spectra of Food Colorants in Solution: Anthocyanins and Curcumin as Case Studies

**DOI:** 10.3390/molecules29184378

**Published:** 2024-09-14

**Authors:** Sara Gómez, Piero Lafiosca, Tommaso Giovannini

**Affiliations:** 1 Classe di Scienze, Scuola Normale Superiore, Piazza dei Cavalieri 7, 56126 Pisa, Italy; sara.gomezmaya@sns.it (S.G.); piero.lafiosca@sns.it (P.L.); 2 Department of Physics, University of Rome Tor Vergata, Via della Ricerca Scientifica 1, 00133 Rome, Italy

**Keywords:** food colorants, curcumin, anthocyanins, QM/MM, MD, absorption spectrum, UV/Vis

## Abstract

We present a comprehensive computational study of UV/Vis absorption spectra of significant food colorants, specifically anthocyanins and curcumin tautomers, dissolved in polar protic solvents, namely water and ethanol. The absorption spectra are simulated using two fully polarizable quantum mechanical (QM)/molecular mechanics (MM) models based on the fluctuating charge (FQ) and fluctuating charge and dipoles (FQFμ) force fields. To accurately capture the dynamical aspects of the solvation phenomenon, atomistic approaches are combined with configurational sampling obtained through classical molecular dynamics (MD) simulations. The calculated QM/FQ and QM/FQFμ spectra are then compared with experiments. Our findings demonstrate that a precise reproduction of the UV/Vis spectra of the studied pigments can be achieved by adequately accounting for configurational sampling, polarization effects, and hydrogen bonding interactions.

## 1. Introduction

UV/Vis spectroscopy is a widely used instrumental technique for identifying and analyzing molecular systems in solution [1,2,3]. UV/Vis spectroscopy is exploited to characterize molecular systems based on their electronic transitions, which are determined by their molecular structure, dynamics, and interactions with an external environment. The technique can be applied to study complex mixtures, reaction kinetics, and molecular binding events [4,5]. Additionally, it is a powerful tool for monitoring the stability and degradation of compounds over time. One notable application is the analysis of food colorants, where understanding absorption properties is critical for ensuring the quality, safety, and appeal of food products [5,6]. The ability to measure the absorption spectra of these compounds allows for the identification and quantification of individual components within complex mixtures [7,8,9]. This is particularly important for food colorants, as their stability and behavior can significantly impact the visual and sensory properties of food products.

In this context, theoretical and computational studies of the absorption properties of molecular systems are crucial for gaining deeper insights into their behavior and interactions [10,11,12,13,14,15]. Theoretical methods can enable the prediction of electronic and structural properties, providing a useful platform to interpret experimental data and guide the design of new compounds with desired physicochemical properties [3,9,15,16,17]. However, as mentioned above, the measured absorption spectrum is substantially affected by the presence of an external environment, such as a solvent [1]. From the computational side, determining how solvent effects affect the electronic structures of molecular systems is particularly challenging. The most common approaches include multi-scale implicit and explicit methods, where the attention focuses on the solute, which is described at the quantum mechanical (QM) level, while the environment is described at a lower level of theory under the assumption that it modifies but not determines the measured spectrum [18,19,20,21,22,23,24]. For systems characterized by strong and specific solute–solvent interactions, such as hydrogen bonding, integrated multi-scale fully atomistic QM/molecular mechanics (QM/MM) approaches have become the gold standard [25,26,27,28,29,30,31,32,33,34]. These approaches allow for a detailed and dynamic representation of solute–solvent interactions, especially when mutual solute–solvent polarization is considered, such as in polarizable QM/MM approaches [30,35,36,37,38,39,40]. The latter provides an accurate yet cost-effective description of the electronic properties of solvated systems, overcoming the limitation of non-polarizable QM/MM methods [14,41].

In this study, we exploit two fully polarizable QM/fluctuating charges (QM/FQ [34]) and QM/fluctuating charges and dipoles (QM/FQFμ [42]) approaches to investigate the UV/Vis spectra of food colorants dissolved in protic polar solvents, such as water and ethanol. The QM/FQ model incorporates a set of fluctuating charges in the MM portion [34], while in QM/FQFμ, an additional set of polarization sources (fluctuating dipoles) is included [42]. Charges (and dipoles) fluctuate as a response to the QM potential (and field) and enter the molecular Hamiltonian to introduce mutual solute–solvent interactions effectively [41,43]. Both approaches are particularly suited for describing the molecular response of systems embedded in polar protic solvents [44,45,46,47,48,49], characterized by specific interactions such as hydrogen bonds. Such fully atomistic methods can be coupled to a dynamical description of the solute–solvent phase-space [41], which is generally sampled by means of classical MD simulations, although more sophisticated methods can also be exploited. In this way, the dynamic aspects of the solvation phenomenon are taken into account.

We apply the procedure to study the UV/Vis spectra of two molecules that have been used in the food industry as colorants, additives, and antioxidant agents [50,51]. In fact, understanding their absorption properties is crucial for their application in the food industry, as these properties influence the color stability and visual appeal of food products. For this reason, these systems have attracted a lot of interest in the literature [52,53,54,55,56,57,58,59,60,61,62,63,64,65]. The first molecule is cyanidin, CYD (3,3′,4′,5,7-pentahydroxyflavylium), which belongs to the broad anthocyanins family. Anthocyanins [66,67,68] are water-soluble natural dyes responsible for the variety of colors (pink, orange, red, violet, and blue) and brightness of many fruits/vegetables (e.g., grapes, berries, and eggplants), flowers/leaves (e.g., pansies, pelargonium, and delphiniums), and food products (e.g., wine, jam, syrup, and preserves). Anthocyanins are, in turn, members of the flavonoid family and can be described as glycosides of polyhydroxy and polymethoxy derivatives of the 2-phenylbenzopyrylium backbone. The aglycon structure formed when the sugar moiety is removed from anthocyanins is called anthocyanidin. The six main anthocyanidins are pelargonidin, peonidin, delphinidin, petunidin, malvidin, and CYD, and by differing in the substitution pattern, they exhibit very diverse coloring features [57,58,68]. The second investigated case is curcumin. Curcumin, the active compound in turmeric (Curcuma longa), is a widely used food coloring since it provides orange hues to bakery products, dairy products, mustard, yogurt, ice cream, and salad dressings [69,70,71]. Curcumin exhibits tautomeric behavior [72,73,74,75,76,77,78,79,80]. For this reason, in this work, we study both enol–keto (EK) and keto–keto (KK) tautomers, whose relative stability depends on several factors, such as solvents, pH, and physical phase [81]. The optical properties of both chromophores (CYD and CUR) and the relative stability of CUR EK–KK tautomers are highly sensitive to environmental factors such as the pH of the solution [82], and are substantially influenced by the solvent, temperature, and substitution pattern, especially the positions and numbers of hydroxyl and methoxy groups and inter- and intramolecular association mechanisms [59].

The paper is structured as follows: our numerical results are discussed, focusing on the conformational study and MD simulations to obtain information on conformations and hydrogen bonding patterns. The final part of the study is dedicated to the discussion of UV/Vis spectra. The computational methodology exploited to calculate the absorption spectra of solvated systems at the QM/FQ and QM/FQFμ levels is recalled and followed by a summary and conclusions.

## 2. Results and Discussion

In this section, we first analyze the conformational landscape of cyanidin as dissolved in water and ethanol. The computed spectra are then discussed in light of the physico-chemical interactions in the solution and compared with the experimental data. A similar analysis is finally reported for curcumin tautomers (EK and KK) in aqueous solution.

### 2.1. Cyanidin Dissolved in Water and Ethanol

#### 2.1.1. MD Analysis and Hydrogen Bonding Patterns

**Figure 1:**
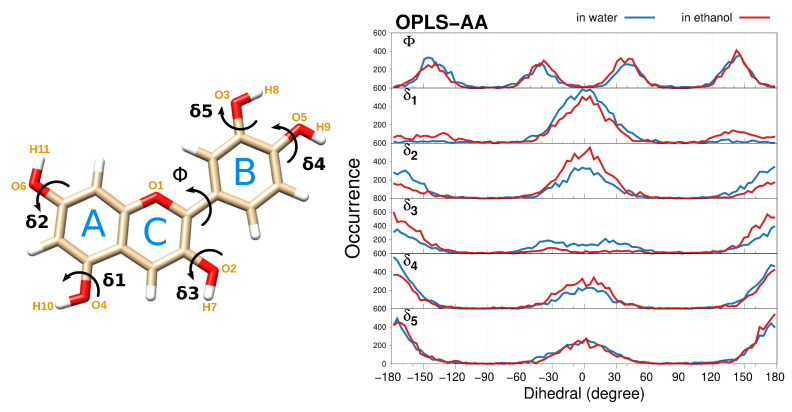
(**left**) CYD molecular structure of labeling adopted in this work. (**right**) Dihedral distribution functions (DDF) of the flexible torsions of CYD solvated in water and ethanol, as calculated from OPLS-AA MD.

Our computational protocol is first applied to the cationic CYD chromophore, which is characterized by a 15-carbon structure consisting of the linkage of two condensed AC aromatic rings (also called benzopyrylium) with a polyphenol cycle (B, also called catechol) through a single bond (see Figure 1, left).

Planarity in CYD is indicative of a good degree of delocalization/conjugation in benzopyrylium that also extends to the 2-phenyl ring [83], which directly reflects on the anthocyanidins absorption spectra (vide infra). Many researchers agree with the quasi-planarity of the molecule [52,56,59,84,85,86,87,88,89], consistent with reported X-ray crystal studies [90]. Figure 1 (right) illustrates the dihedral distribution functions (DDFs) for the main torsions of CYD as dissolved in water (blue) and ethanol (red). The distribution of the Φ dihedral, which defines the position of the polyphenol ring with respect to the fused rings, is shown in the top panel. Such a dihedral angle is directly related to the planarity of the molecule. By using OPLS-AA FF, CYD does not undergo strong structural modifications, and the four groups of conformers (as related to Φ dihedral angle) sampled during the MD agree with the quasi-coplanarity of the benzopyrylium and the 2-phenyl rings mentioned above. Remarkably, our DDFs of Φ resemble the four-peak distributions resulting from accurate ab initio MD simulations for cyanidin-3-glucoside (cyanin) in explicit water [65]. Conversely, the set of parameters derived with the GAFF force field induces a partial distortion of the CYD molecule and a remarkable rotation of the catechol ring with dihedral values of around −90º and 90º in water and ethanol, respectively, as seen in Figure S1 in the Supplementary Material (SM). This demonstrates that the geometries (and consequently the optical properties) of natural dyes like CYD may dramatically depend on the choice of the FF.

The behavior of $\delta_{1}$–$\delta_{5}$ dihedrals defining the orientation of each hydroxyl group of CYD along the whole trajectory is also displayed in Figure 1. For both protic solvents, hydroxyl groups are very flexible during the simulation time but preferences are seen for those CYD conformers that keep the –OH portion in the plane ($\delta_{i}$ = 0º and 180º), even though large amplitude motions that stem from the interactions with the solvent are found.

**Figure 2:**
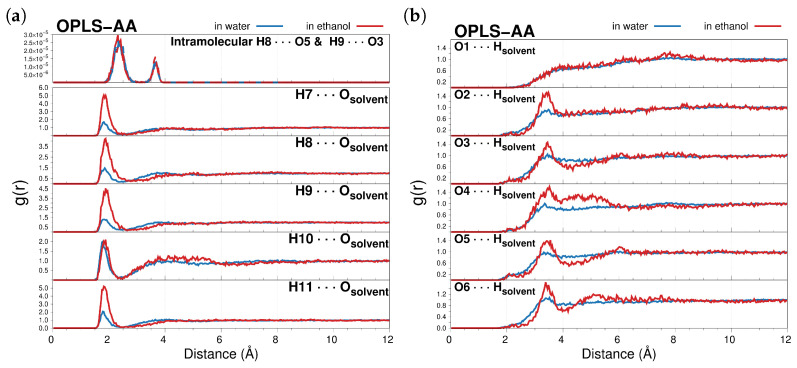
Radial distribution functions g(r) of intramolecular (top) and intermolecular interactions between selected H_*i*_ atoms of CYD and the solvent oxygen atoms (H_*i*_⋯ O_solvent_, (**a**)) and between the O_*i*_ atoms of CYD and the solvent hydrogen atoms (O_*i*_ ⋯ H_solvent_, (**b**)). All RDFs are computed along the OPLS-AA MD trajectories.

The specific solute–solvent interactions are studied in terms of the H-bonding sites. CYD has five H-donor (O2–O6, to which H7–H11 are attached in Figure 1) and six H-acceptor (O1–O6 in Figure 1) sites. Radial distribution functions (RDFs) between those selected sites of CYD and water/ethanol molecules are shown in Figure 2.

By analyzing the RDFs, it is possible to obtain a deeper understanding of the CYD atoms that are more prone to be involved in HBs with the solvent. The position of the RDFs maxima and the corresponding running coordination numbers (RCNs) resulting from their integration over the first solvation shell are listed in Table 1 and give ≈5 water and ≈4 ethanol molecules close to CYD and mostly located around the CYD hydroxyl groups (see also Figure S2 in the Supplementary Materials). Notice that peaks in the RDF plots when the CYD oxygens act as acceptors (see Figure 2b) barely appear in the second solvation shell, which is in agreement with the findings reported in ref. [64]. This can be interpreted as a case where the dye acts better as a proton donor in solution. Furthermore, the RDF peaks for H_*i*_⋯ O_solvent_ pairs in ethanol are always found at slightly longer distances with respect to the water ones, thus highlighting stronger interactions between CYD⋯solvent in an aqueous solution. RCNs for H8 and H9 (see Table 1) in conjunction with the intramolecular atom–atom pair correlation functions shown in the top panel of Figure 2a suggest that the competitive intra- and intermolecular interactions are inclined towards contact between the solvents. Therefore, even weak interactions between CYD and solvent molecules or, in less diluted solutions, between CYD molecules as well, can alter the equilibrium value of the Φ dihedral angle and influence CYD hues [59]. Results obtained using the GAFF FF provide a picture of the chromophore interacting more weakly with both solvents (see Figures S3 and S4 and Table S1 in the Supplementary Materials).

The CYD conformers explored during the simulations can be further filtered to extract representative structures and inspect their properties. A detailed look at the top panel of Figure 1 and the temporal evolution of the Φ dihedral angle depicted in Figure S5 in the Supplementary Materials confirms that despite the high conformational heterogeneity of the CYD structures along the simulations, they can be clusterized through a dihedral-based cluster analysis in four groups, similarly to ref. [65]: 90º <Φ< 180º—first group, 0º <Φ< 90º—second group, −90º <Φ< 0º—third group, and −180º <Φ<−90º—fourth group. The central structure of each cluster (see Figure S5 in the Supplementary Materials) is then extracted as the most representative one, and the population is derived from the number of members of each group. The final representative structures and the associated populations are displayed in Figure S6 in the Supplementary Materials. Note that given the distribution of Φ originated from the MD with GAFF (Figure S1, top panel), RMSD-based clustering using the GROMOS clustering tool [91] is instead used, showing significant differences with respect to OPLS-AA clustered structures, as expected (see Figure S7 in the Supplementary Materials).

#### 2.1.2. Absorption Spectra

From the DDFs and clustering analysis presented before, it is confirmed that many CYD conformers coexist in solution, and each of their contributions should be taken into account for determining the electronic absorption spectrum of CYD in a chosen solvent. As pointed out in the introduction, calculating spectroscopic properties with our robust protocol allows obtaining an averaged spectrum derived from a set of snapshots extracted from the MD simulations.

**Figure 3:**
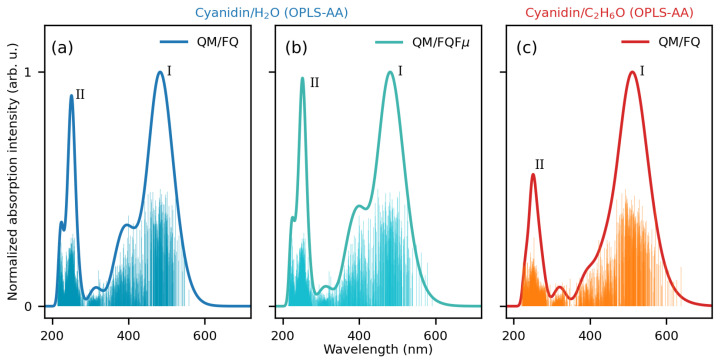
Stick and convoluted (normalized) spectra of solvated CYD in (**a**) water at the QM/FQ level; (**b**) water at the QM/FQFμ level; (**c**) ethanol at the QM/FQ level. All spectra are computed using 200 frames extracted from OPLS-AA MD runs.

Figure 3 displays the final modeled spectra of CYD in water and ethanol, collected after averaging 200 individual spectra corresponding to instantaneous configurations of the fully solvated system. The convergence analysis of the computed spectra as a function of the number of snapshots is reported in Figures S8 and S9 in the Supplementary Materials. As stated above, the absorption spectrum in aqueous solution is computed at the QM/FQ and QM/FQFμ levels, while that in ethanol is calculated at the QM/FQ level only. Figure 3 reports the computed stick spectra (raw data) for each snapshot. The spread of the sticks clearly shows the natural origin of the inhomogeneous band broadening (convoluted line), which is automatically taken into account by the dynamic sampling of the solute–solvent phase space. Notably, the sticks in ethanol feature a broader distribution and, in turn, a broader absorption band with respect to the spectra in aqueous solution.

In the whole spectral region from 200 to 700 nm, the convolution of the sticks leads to QM/FQ and QM/FQFμ spectra of CYD in water that are dominated by two main sharp peaks: peak I at λ = 483 nm (2.57 eV) and λ = 481 nm (2.58 eV), respectively, and peak II near 250 nm (4.96 eV). There is also a prominent shoulder of the peak I, around λ = 400 nm (3.10 eV). While QM/FQ and QM/FQFμ are remarkably similar, some minor discrepancies can be appreciated, especially with regard to the relative intensities of the peaks. In fact, at the QM/FQFμ, the main peaks (I and II) present almost the same intensities, while at the QM/FQ level, peak I is higher than peak II. Vice versa, the shoulder at about 400 nm is predicted with larger intensities by QM/FQFμ than QM/FQ. The spectrum of CYD in ethanol (Figure 3c) also consists of two bands: one high-intensity absorbance band in the visible region at λ = 512 nm (2.42 eV) and a lower-intensity absorbance band at λ = 251 nm (4.94 eV). Additionally, our simulations predict a weaker feature in both solvents at λ≈ 320 nm. GAFF counterparts are shown in Figure S12 in the Supplementary Materials. While the main peaks (I and II) can be recognized, the simulated spectra using the structures extracted from the GAFF trajectory substantially differ from those reported in Figure 3. This is due to the completely different conformational sampling provided by the GAFF force field, whose structures are mainly characterized by strongly distorted Φ (≈90º and −90º—see Figure S13 in the Supplementary Materials). As a consequence, the electron delocalization is hampered in the entire 2-phenylbenzopyrylium core, which acts as a unique π-conjugated system.

**Figure 4:**
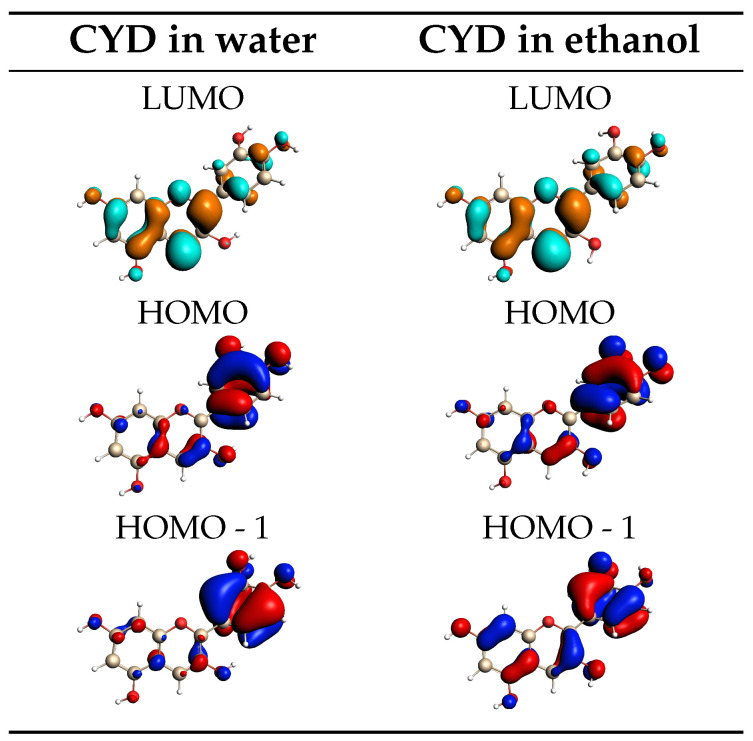
QM/FQ MOs involved in the vertical transitions of CYD in water (**left**) and in ethanol (**right**) computed for a representative structure extracted from OPLS-AA MD runs.

To characterize the electronic transitions, relevant molecular orbitals (MOs) of CYD in water and in ethanol are shown in Figure 4, as calculated for the most representative structure extracted from the trajectory (vide infra). The frontier orbitals of the system have a π character and are mainly delocalized over the benzopyrylium and catechol rings since the anthocyanidin core is a fully delocalized π-conjugated system, which is why these compounds can absorb visible light [68]. An MO rationalization of the main band that appears in the experimental spectra indicates that it is determined by the $S_{0}\to S_{1}$ transition, which is essentially a HOMO-LUMO transition of $\pi \to \pi^{*}$ character, accounting for around 95% and 96% of the total oscillator strength in water and ethanol, respectively. The shoulder of the maximum absorption band, located at around 400 nm, corresponds to the transition to the second excited state ($S_{2}$) and is mainly assigned to the HOMO-1 → LUMO transition. This assignment of the electronic transitions is in line with the findings reported in other works [56,60,61,88]. The similarities found in the MOs and hydration patterns of CYD in water and ethanol are thus reflected in the same shape of the spectral profiles. It is also worth remarking that, by using the structures extracted from GAFF MD runs, as expected, the outlined picture is completely different for the aforementioned discrepancies in the sampling of the solute conformational space (see Figure S13 in the Supplementary Materials).

**Figure 5:**
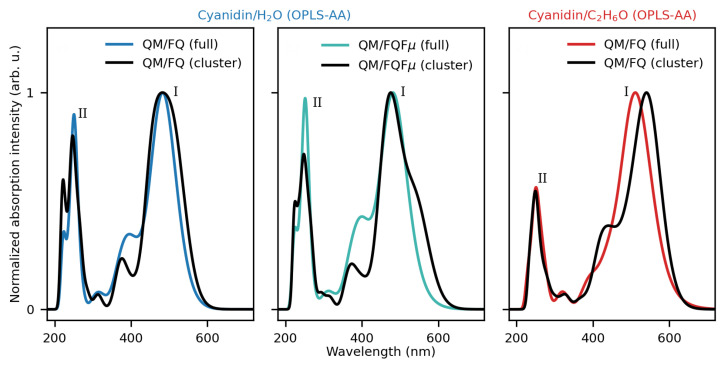
UV/Vis spectra of solvated CYD computed using all frames (“full”) and the representative structures resulting from clustering (“cluster”) of the OPLS-AA MD runs.

An interesting question arises as to whether the predominant spectral attributes of CYD in solution can be grasped by averaging the spectra of the main conformers using the contributions given by their relative population, as already investigated for other systems in the literature [92,93,94,95]. Computed spectra via the full set of configurations and exploiting those of the most populated conformers are displayed in Figure 5 (a similar analysis for GAFF clusters is reported in Figure S14). We find that for CYD in water, with the full set (“full”) or the clusterized set (“cluster”), the positions of the maxima (peaks I and II) are quite close in both QM/FQ and QM/FQFμ approaches, accompanied by in a slight decrease in the absorption intensity of the shoulder. Using clusters in the ethanol case results in a red (bathochromic) shift of the main band (I) while leaving the position and shape of band II almost unaffected. Even though the procedure of using clusters is cheaper and produces spectra that look similar, the broadening of the bands and the emergence of key spectral properties may require information from larger groups of configurations, potentially yielding quantitative disagreement, as shown for CYD in ethanol.

Let us now move to compare our computed results with the experimental results recovered from ref. [96]. From the experimental point of view, anthocyanidins generally present two main absorption bands in the UV/visible spectrum. The first is observed in the 270–280 nm range (band II), while the second is in the 465–560 nm range (band I) both in alcoholic solution and in the presence of hydrochloric acid. A third small-intensity band peaking at 310–360 nm has also been reported [58,85]. Although the three bands of anthocyanidins are all captured in our modeled spectra, we focus our attention on the main peak located in the visible region. A critical assessment of available spectroscopic data for CYD and its glycosylated compound, namely cyanin, showed that several independent measurements of $\lambda_{max}$ are available in hydrochloric acid in methanol, water, and ethanol as solvents. Likewise, depending on the approaches in the modeling, diverse results from calculations are available as well. The gathering of such data is listed in Table S3 in the Supplementary Materials. Summarizing the findings reported in ref. [96], the absorption spectrum for the CYD (and cyanin molecule) in water solution at room temperature displays a broad band all over the visible range, characterized by two prominent peaks at 512 and 429 nm. Regardless of the solvent, our results are in good agreement with experiments both in the shape and in the position of the lowest energy peaks when configurations come from MDs with OPLS-AA FF (the molecule is quasi-planar).

**Figure 6:**
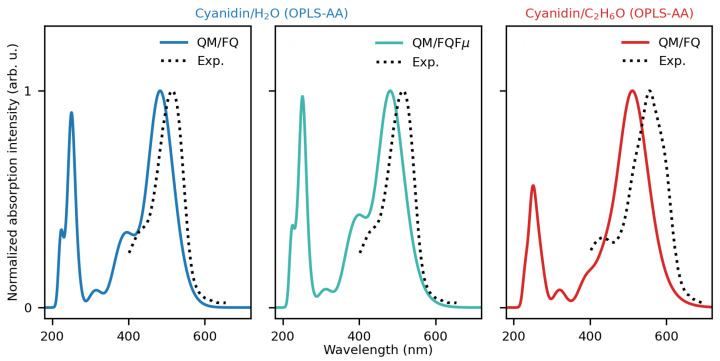
Computed and experimental UV/Vis spectra of solvated CYD. QM/MM frames are extracted from OPLS-AA MDs. Experimental data are taken from ref. [96].

When compared against the measured spectrum (see Figure 6), QM/FQ and QM/FQFμ approaches give qualitatively and quantitatively similar spectral results for CYD in water. However, the relative intensity between the main band and its shoulder is better captured by QM/FQFμ, highlighting the relevance of anisotropic solute–solvent interactions. Changing the solvent of CYD imparts evident changes in the spectra, particularly in the visible portion. First, by passing from water to ethanol, a red-shift of the entire visible band of 30 nm (0.15 eV) is observed, in particularly good agreement with the experiment (40 nm, 0.20 eV—error∼1 kcal/mol). Such an agreement deteriorates if an implicit QM/COSMO approach is exploited, for which a solvatochromic shift of just 0.03 eV is obtained (see Figure S16 in the Supplementary Materials). We remark that solvatochromic shifts, being calculated as energy differences, reduce the systematic error associated with the particular choice of the DFT functional/basis set [97]. Second, the main band in ethanol becomes broader, and third, detected as an easily recognizable shoulder in water, decreases in intensity, turning into an almost imperceptible feature. The overall agreement with the experiment, also considering the small difference between the computed and experimental excitation energies, is excellent, especially considering that vibronic effects have not been considered in our simulations [88].

By considering the GAFF MD, CYD absorption spectra in water and in ethanol exhibit further features that strikingly contrast with the experimental observations (see Figure S15 in the Supplementary Materials). These discrepancies are again a consequence of the fact that in frames extracted from the GAFF MD, the phenyl moiety is twisted (Φ close to ±90°).

### 2.2. Curcumin Tautomers in Aqueous Solution

**Figure 7:**
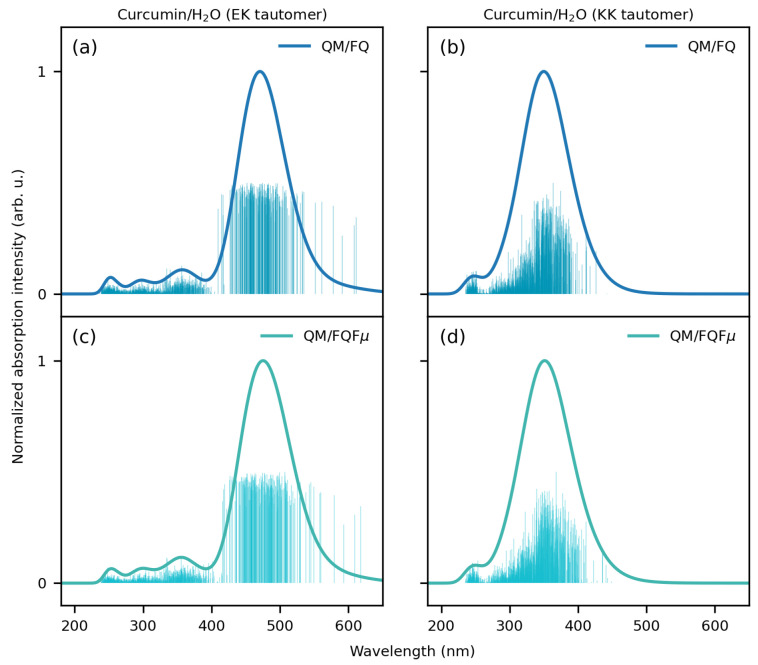
Stick and convoluted (normalized) spectra of solvated EK (**left**) and KK (**right**) tautomers of curcumin dissolved in water as computed at the QM/FQ (**a**,**b**) and QM/FQFμ (**c**,**d**) levels.

We now move to curcumin tautomers dissolved in aqueous solution. As stated above, the MD trajectory is recovered from ref. [98], where the MD analysis is amply discussed. While the EK structure is mainly planar due to its conjugated structure, the KK structure is highly flexible, especially considering the dihedral angle involved in the enol–keto tautomerism. The different molecular electronic structure of the two tautomers reflects on the computed UV/Vis spectra, which are graphically depicted in Figure 7 together with the associated raw data (stick spectra).

As for CYD, solvent effects on curcumin EK and KK spectra are modeled using the polarizable QM/FQ and QM/FQFμ approaches. QM/MM calculations are performed on 200 uncorrelated snapshots extracted from MD runs. The number of uncorrelated frames guarantees the convergence of the computed spectra (see Figures S17 and S18 in the Supplementary Materials).

In Figure 7, the stick spectra computed for EK (Figure 7a,c) and KK (Figure 7b,d) tautomers at the QM/FQ and QM/FQFμ levels UV/Vis show high variability in both the intensity and energy associated with each snapshot. This is directly related to the fact that our approach accurately considers the dynamical aspects of the solvation phenomenon through the classical MD simulations. It is also worth pointing out that such variability is due to the dynamical exploration of the solvent and solute phase-space sampling. As for CYD, the main consequence is the automatic inclusion of inhomogeneous band-broadening (see convoluted blue lines in Figure 7).

**Figure 8:**
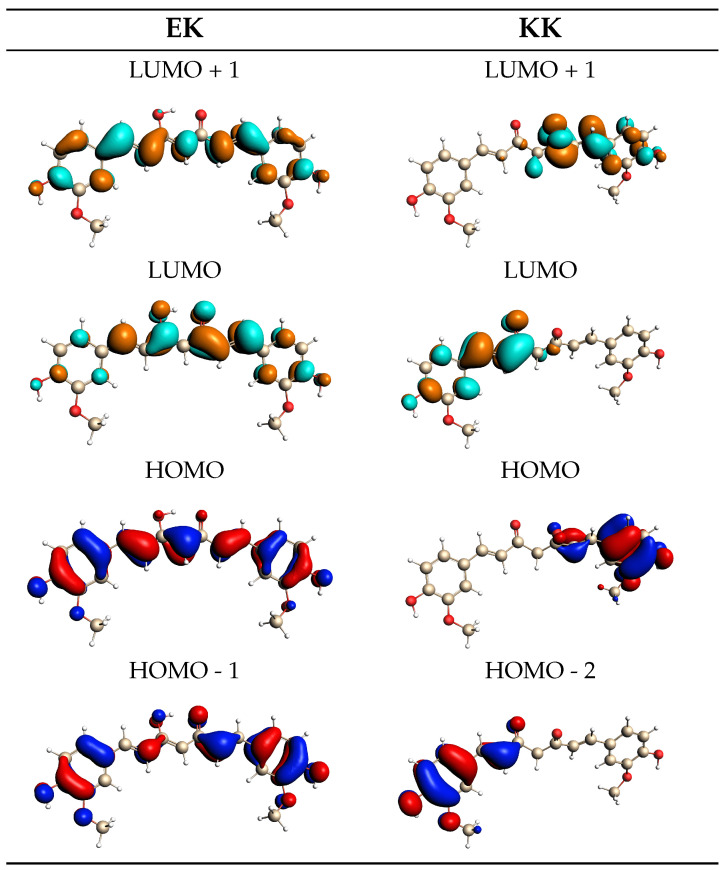
QM/FQ MOs involved in the vertical transitions of the EK (**left**) and KK (**right**) tautomers of curcumin computed for a representative snapshot.

The convoluted QM/FQ and QM/FQFμ EK and KK UV/Vis spectra are dominated by a main bright band located at about 470 and 350 nm, respectively. The two fully polarizable approaches provide similar computed spectra (see also Table S4 in the Supplementary Materials where the absorption wavelengths are reported). This demonstrates that in this case, specific solute–solvent interactions, electrostatics, polarization, and dynamic effects overcome the anisotropic solute–solvent interactions accounted for by QM/FQFμ. Note, however, that this is an average effect because, for each snapshot, QM/FQ and QM/FQFμ yield a diverse UV/Vis spectrum. To analyze the nature of the electronic transition, in Figure 8, the EK and KK MOs involved in the excitation are graphically depicted as computed for a representative snapshot extracted from the MD runs. For both EK and KK tautomers, the lowest and most intense transition predicted by all considered approaches has a π–$\pi^{*}$ character. This can be appreciated by the involved MOs: for EK, the transition is mostly (94%) from HOMO to LUMO, with the remaining percentage concerning also HOMO-1 → LUMO+1. For the KK tautomer, the signal is mainly (64%) due to a HOMO → LUMO+1 transition, but there is also an important contribution (21%) from HOMO-2 to LUMO. The MOs depicted in Figure 8 highlight the different natures of the electronic structures of the two tautomers. In fact, the MOs involved in the main electronic transition of the KK tautomer are characterized by a significant charge-transfer and electron delocalization character, thus validating the use of a range-separated functional such as CAMY-B3LYP.

**Figure 9:**
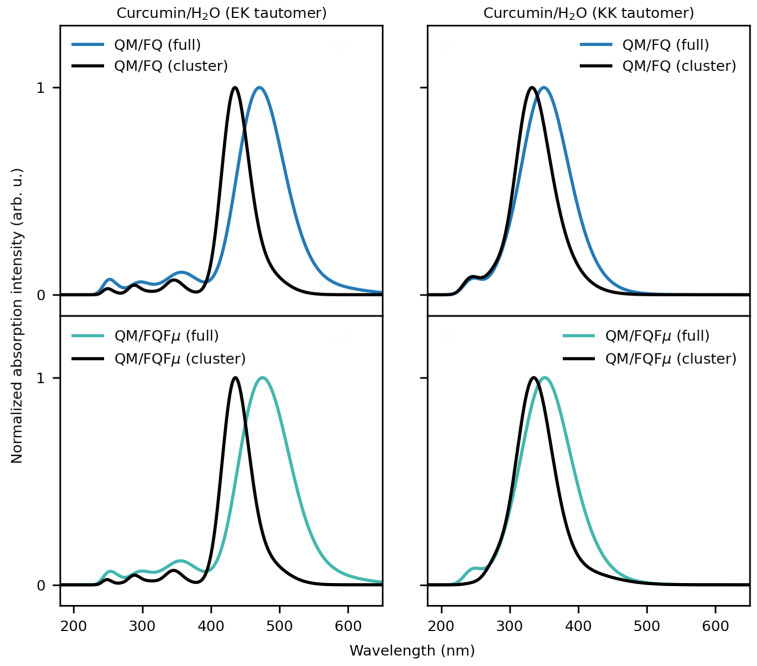
UV/Vis spectra of solvated EK and KK tautomers computed using all frames (“full”) and the representative structures resulting from clustering (“cluster”).

Similarly to CYD in aqueous solution, also for EK and KK CUR tautomers, we investigate whether the computed spectra can be qualitatively and quantitatively obtained by averaging the spectra of the main conformers of both tautomers in aqueous solution as weighted by the associated populations (see Figure S19 in the Supplementary Materials). The resulting computed spectra (“cluster”) are compared with those obtained by exploiting the full set of configurations (“full”) in Figure 9. Qualitatively, the spectra are correctly reproduced, as they are both characterized by a main bright absorption band. However, the absorption maxima computed using the “cluster” structures are systematically shifted at higher energies than the reference “full” spectra. Therefore, our findings highlight that clustering techniques that can be exploited to reduce the cost of QM/MM calculations must be carefully validated, as they might provide incorrect quantitative results. In addition, as also noticed for CYD, the band broadening, and in particular its inhomogeneous character, requires a larger group of configurations.

**Figure 10:**
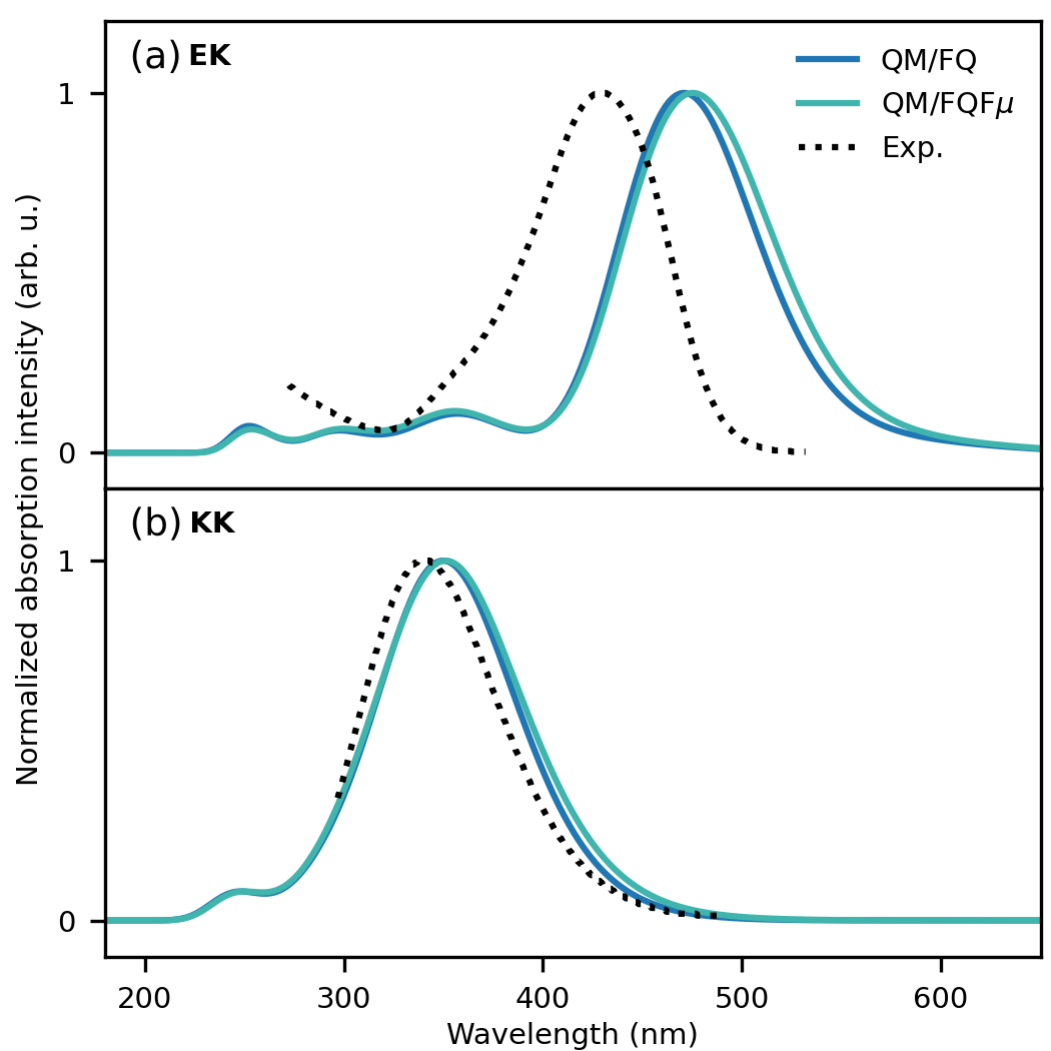
Computed and experimental UV/Vis spectra of solvated EK (**a**) and KK (**b**) tautomers. Experimental data are taken from ref. [98].

We now move to the comparison with the experimental spectra. EK and KK computed QM/FQ and QM/FQFμ spectra are reported in Figure 10, together with their corresponding experimental counterparts, which are recovered from ref. [98]. Both EK and KK experimental spectra are dominated by a single vertical transition placed at about 429 and 340 nm, respectively. Such a shift is probably due to the exploited choice of the DFT functional/basis set [98]. The broadening of both curves is almost homogeneous, reflecting the fact that vibronic couplings do not play a substantial role. In the case of EK (see Figure 10a), the experimental maximum lies at about 429 nm. The theoretical approaches predict a red-shifted maximum, which is placed at 471 and 475 nm for QM/FQ and QM/FQFμ methods, respectively. Although for EK the MD sampling predicts one main conformer to be populated (see Figure S19), the fluctuations around the equilibrium position are crucial to correctly accounting for conformational effects, as reflected by a very good reproduction of the band broadening. Such an inhomogeneous broadening cannot be obtained by exploiting an implicit, static QM/COSMO approach, which, however, provides absorption spectra in good agreement with the experiments (see Figure S20 in the Supplementary Materials). In the case of the KK tautomer (see Figure 10b), the QM/FQ and QM/FQFμ computed spectra are almost superimposed on the experiment. Our findings thus highlight that an excellent agreement with the experimental results can be achieved by accurately sampling the solute–solvent phase-space and by properly incorporating specific solute–solvent interactions, as well as mutual solute–solvent polarization effects.

## 3. Materials and Methods

To calculate the absorption UV/Vis spectra of solvated systems using QM/MM approaches, we exploit the procedure reported in refs. [41,46]. Such a protocol consists of several steps that are needed to ensure that the various physicochemical interactions between the target molecule and the solvent are consistently considered and transferred to computed spectra.

1. *Definition of QM/MM boundaries:* The parts of the system to be treated as the “solute” and as the “solvent” need to be delineated. For a solution, the solute is described at the QM level, while the solvent is treated at the MM level. The boundary between these two regions (i.e., the QM/MM boundary) is thus defined accordingly.
2. *Dynamical sampling:* To account for the dynamical aspects of the solvation phenomenon, the solute-solvent phase space must be sampled. In this work, we resort to classical non-polarizable MD simulations, which have been shown to be particularly reliable when combined with QM/MM calculations for computational spectroscopy [41]. For curcumin tautomers in aqueous solution, we borrow the trajectories obtained in ref. [98], where two 10 ns classical MD simulations were performed by treating each curcumin tautomer with the GAFF force field while describing water molecules by means of the TIP3P force field.
  We then study CYD dissolved in water and ethanol. CYD is the sugar-free counterpart of the cyanidin-3-glucoside pigment. In this study, we do not consider the sugar portion as it does not directly participate in the optical transitions on the conjugated core [60,65]. CYD is known to present different behaviors depending on the pH range. Here, we study it in the *flavylium*-charged state, which is the most stable under acidic conditions (pH < 3) and perform MD simulations of CYD in solution, namely, water and ethanol, plus a Cl− counterion to neutralize the system. To parametrize the chromophore, we first analyze its main conformers. Initial CYD structures are generated using the AMS Conformers tool [99], then optimize it at the density functional tight-binding (DFTB) level [100] to be finally scored by employing the CAMY-B3LYP/TZP level in the ADF engine [99,101]. The best-ranked conformer (planar) is used to obtain parameters using two force fields, namely OPLS-AA and GAFF, exploiting the LigParGen [102,103,104] and acpype web servers [105,106], respectively. In conjunction with TIP3P parameters, GAFF was used in ref. [65], while OPLS-AA was used in ref. [64]. Electrostatics are refined using the CM5 atomic charges [107] computed at the CAMY-B3LYP/TZP level.
  CYN is fully solvated in TIP3P water [108] and separately in ethanol, using cubic cells of 62 Å edge accommodating 7500 and 2500 solvent molecules, respectively, in periodic boundary conditions. Initial velocities are assigned according to the Boltzmann distribution at 300 K. For both charge–charge and van der Waals terms, a cutoff of 11 Å is used, while the particle mesh Ewald method is employed to account for long-range electrostatics. MDs are run in an NPT ensemble after NVT and NPT equilibration stages, which last for 1 and 2 ns, respectively, with a V-rescale [109] thermostat and Berendsen barostat [110]. In the NPT production stage, keeping temperature (300 K) and pressure (1 atm) constant, an integration time step of 2 fs is employed for a total simulation time of 30 ns. All MD simulations are performed using the GROMACS 2020.4 software [111]. The analyses of the trajectories are performed with the TRAVIS package [112,113].
3. *Extraction of structures:* Two hundred uncorrelated snapshots are extracted from each MD run and employed for the subsequent QM/MM calculations. The snapshots are shaped as spherical “droplets” with a radius of 20 Å for curcumin tautomers, and 18 Å for CYD (see Table 2 for a graphical depiction). The total number of snapshots is chosen to guarantee the convergence of the final computed spectrum.
4. *Polarizable QM/MM calculations*: On each extracted snapshot, the absorption spectrum is calculated at the fully polarizable QM/FQ and QM/FQFμ levels. In a QM/MM approach, the total energy of the system can therefore be written as follows [29]:
  (1) $$E=E_{\mathrm{QM}}+E_{\mathrm{MM}}+E_{\mathrm{QM}/\mathrm{MM}}^{\mathrm{int}},$$
  where $E_{\mathrm{QM}}$ and $E_{\mathrm{MM}}$ are the energies of the QM and MM portions, while $E_{\mathrm{QM}}^{\mathrm{int}}$ defines the interaction energy between the two layers. In the QM/FQ and QM/FQFμ approaches, the MM atoms are endowed with a fluctuating charge (FQ) and dipole (FQFμ), which can dynamically respond to the QM density ($\rho_{\mathrm{QM}}$). Therefore, the QM/MM interaction $E_{\mathrm{QM}}^{\mathrm{int}}$ can be specified in terms of the Coulomb law between the two portions as follows [41,42,46]:
  (2) $$E_{QM/FQ}=\sum_{i}^{N_{q}}q_{i}\left(\rho_{\mathrm{QM}}\right)V_{i}\left(\rho_{\mathrm{QM}}\right),$$
  (3) $$E_{QM/FQF\mu }=\sum_{i}^{N_{q}}q_{i}\left(\rho_{\mathrm{QM}}\right)V_{i}\left(\rho_{\mathrm{QM}}\right)-\sum_{j}^{N_{\mu }}\mu_{j}\left(\rho_{\mathrm{QM}}\right)\cdot E_{j}\left(\rho_{\mathrm{QM}}\right),$$
  where $q_{i}$ and $\mu_{i}$ are the charge and dipole of the *i*-th atom, while $V_{i}\left(\rho_{\mathrm{QM}}\right)$ and $E_{i}\left(\rho_{\mathrm{QM}}\right)$ are the electric potential and field due to the QM density calculated on the same MM atom. The charges and dipoles are polarized by the QM density and are obtained by solving the following linear equation [46]:
  (4) $$\left(\begin{array}{ccc} T^{qq} & 1_{\lambda } & T^{q\mu }\\ 1_{\lambda }^{†} & 0 & 0\\ T^{q\mu^{†}} & 0 & T^{\mu \mu } \end{array}\right)\left(\begin{array}{c} q\\ \lambda \\ \mu \end{array}\right)=\left(\begin{array}{c} -\chi \\ Q_{\mathrm{tot}}\\ 0 \end{array}\right)+\left(\begin{array}{c} -V(D)\\ 0\\ E(D) \end{array}\right),$$
  where $1_{\lambda }$ contains the Lagrangian (λ) blocks used to ensure that the total charge of each MM molecule is kept fixed to $Q_{\mathrm{tot}}$. $T^{qq}$, $T^{qm}$, and $T^{\mu \mu }$ are the charge–charge, charge–dipole, and dipole–dipole interaction kernels defining the interactions between the polarization sources. Such kernels are defined in terms of atomic chemical hardnesses η and polarizabilities α, which, together with the atomic electronegativities χ are the parameters defining FQ (χ,η) and FQFμ (χ,η,α) force fields. Note that the FQ linear system is obtained by discarding rows/columns involving dipoles in Equation (4).
  In this work, the QM region is defined at the density functional level (DFT) level. The Kohn–Sham matrix $h_{KS}\left(r\right)$ is thus modified by considering the external embedding potential (${\hat{v}}^{emb}$) generated by the MM polarization sources (charges and dipoles) as follows [114]:
  (5) $$h_{KS}\left(r\right)=h_{KS}^{0}\left[\rho \left(r\right)\right]+{\hat{v}}^{emb}\left(r\right),$$
  (6) $${\hat{v}}^{emb}\left(r\right)=q^{†}V_{QM}\left(r\right)-\mu^{†}E_{QM}\left(r\right).$$
  By using Equation (5) in combination with Equation (4), mutual solute–solvent polarization effects are consistently introduced.
  To calculate the absorption spectra of solvated systems, we exploit the linear response time-dependent DFT (TDDFT) [115] extension of the QM/FQ and QM/FQFμ methods (see ref. [44,46]). Within this approach, the polarization sources of the MM portion dynamically respond to the transition density of the QM portion, consistently accounting for polarization effects in the linear response regime.
  All QM/MM calculations are performed using the ADF [101] engine within the Amsterdam Modeling Suite (AMS) v. 2024.1 [99]. The QM part is treated by exploiting the CAMY-B3LYP density functional [116,117] combined with the TZP basis set [118]. TDDFT calculations are performed requesting 10 excited states. Solvent molecules within the MM region are described at the FQ and FQFμ force field using the parameters reported in ref. [97] (FQ: water and ethanol) and [42] (FQFμ: water).
5. *Extraction of spectra and comparison with experiments*: The spectra obtained for each snapshot are extracted and averaged to produce final spectra. In particular, each spectrum is convoluted using a Gaussian-type function with a full width at half maximum (FWHM) of 0.3 eV for CYD and EK tautomer, while 0.6 eV is used for the KK tautomer in agreement with ref. [98]. The final computed spectra are compared with the available experimental data. For the sake of comparison, implicit QM/COSMO [119] calculations have also been performed (see Sections S1 and S2 in the Supplementary Materials for further details).

## 4. Conclusions

In this study, we present a detailed computational study of the UV/Vis absorption spectra of significant food colorants, specifically cyanidin from the anthocyanins family and curcumin tautomers dissolved in water and ethanol. To accurately describe solvent effects on absorption spectra, we rely on a computational protocol that consistently accounts for the dynamic aspects of the solvation phenomenon through a classical MD simulation of each dye dissolved in solution. In this way, the solute–solvent phase space is sampled, and specific solute–solvent interactions are taken into account in the determination of the solute conformers. The classical MD simulation is combined with fully atomistic polarizable QM/MM approaches to physically describe solute–solvent effects in the determination of the electronic structure and transitions of the solute.

Our results for CYD show the necessity of an accurate sampling of the solute–solvent phase-space to obtain reliably computed spectra in agreement with the experimental findings. For the present case study, we have shown that the OPLS-AA force field overperforms GAFF, providing sampling of the solute structures in agreement with ab initio MD. Furthermore, the computed spectra for EK and KK tautomers in aqueous solution confirm that negligible discrepancies between QM/FQ and QM/FQFμ are reported. For both systems, the agreement with the experimental data is, remarkably, particularly good. This highlights that, for the considered systems, the accurate sampling of the solute–solvent phase-space and the physically consistent treatment of the specific solute–solvent interactions, also accounting for mutual solute–solvent polarization effects, is crucial to yield computed results consistent with experiments. On the other hand, the effect of including anisotropic QM/MM interactions as in the case of QM/FQFμ seems to be minor on the average computed spectra, mostly affecting secondary spectral features, although the same does not apply to the single representative structures extracted from the MD simulation.

Lastly, our findings indicate that clustering techniques may be an effective tool for reducing the computational cost associated with QM/MM approaches, which can require hundreds or even thousands of snapshots to achieve convergence [43]. However, it is crucial to rigorously validate these methods for the specific systems under investigation, as the agreement between the converged spectrum and that obtained from the clustered structures can sometimes be qualitatively and quantitatively inaccurate, as observed in the cases of CYD in ethanol and CUR in water. Nonetheless, clustering techniques hold significant promise in this field and have the potential to impact the efficiency of computational QM/MM studies.

## Figures and Tables

**Table 1:** Position of the first peak in the RDFs and running coordination number (RCN) between the hydroxyl hydrogens in CYD (H_*i*_, *i* = 7–11) and the solvent oxygen atoms (O_solvent_, see also Figure 2a). The data are extracted from OPLS-AA MD runs.

	In Water		In Ethanol	
	**Max at (Å)**	**RCN**	**Max at (Å**)	**RCN**
H7⋯O_solvent_	1.82	0.99	1.82	0.87
H8⋯O_solvent_	1.85	0.87	1.88	0.94
H9⋯O_solvent_	1.82	1.02	1.88	0.94
H10⋯O_solvent_	1.78	0.97	1.88	0.34
H11⋯O_solvent_	1.78	1.02	1.82	0.86

**Table 2:** Example of snapshots of CYD dissolved in water and ethanol, and KK and EK tautomers of curcumin dissolved in water.

CYD in Water	CYD in Ethanol	KK in Water	EK in Water
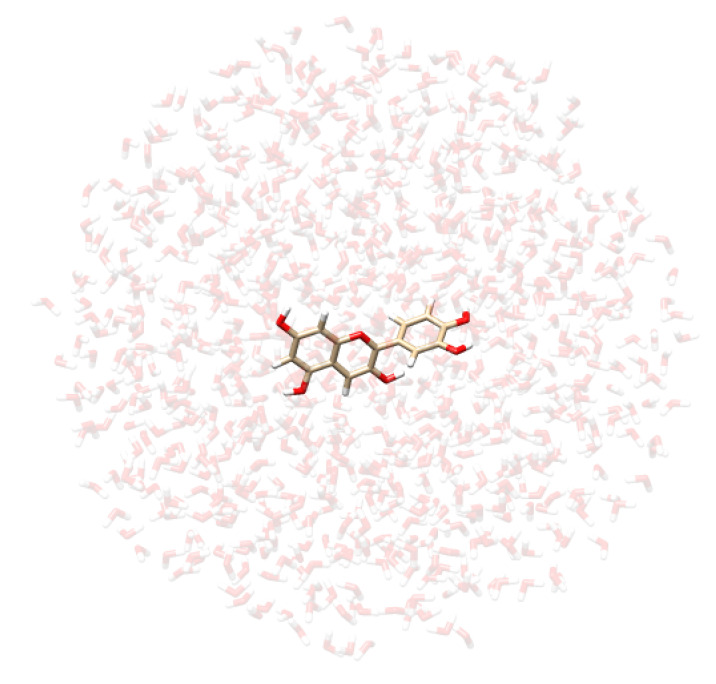	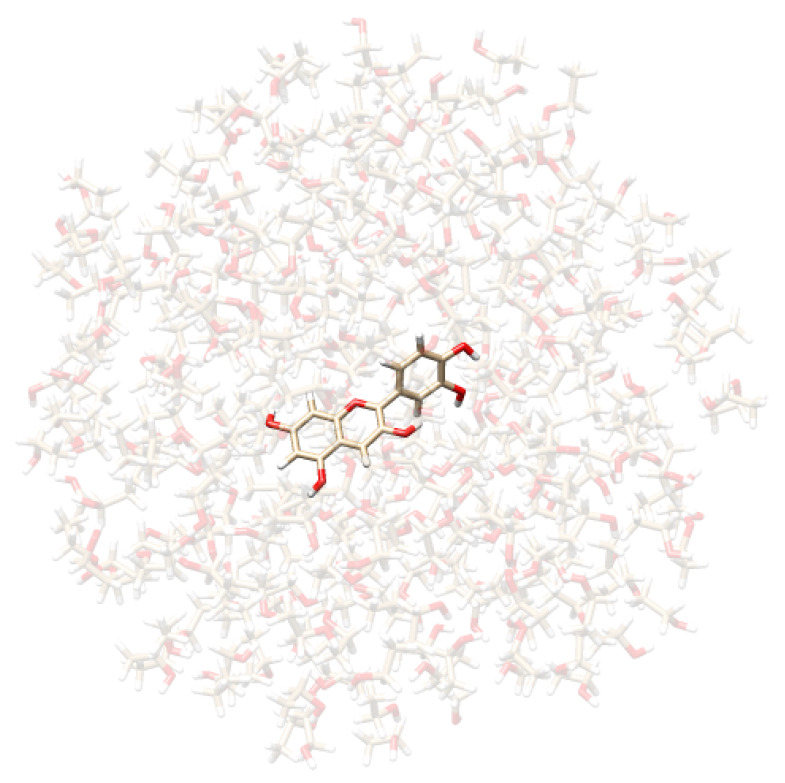	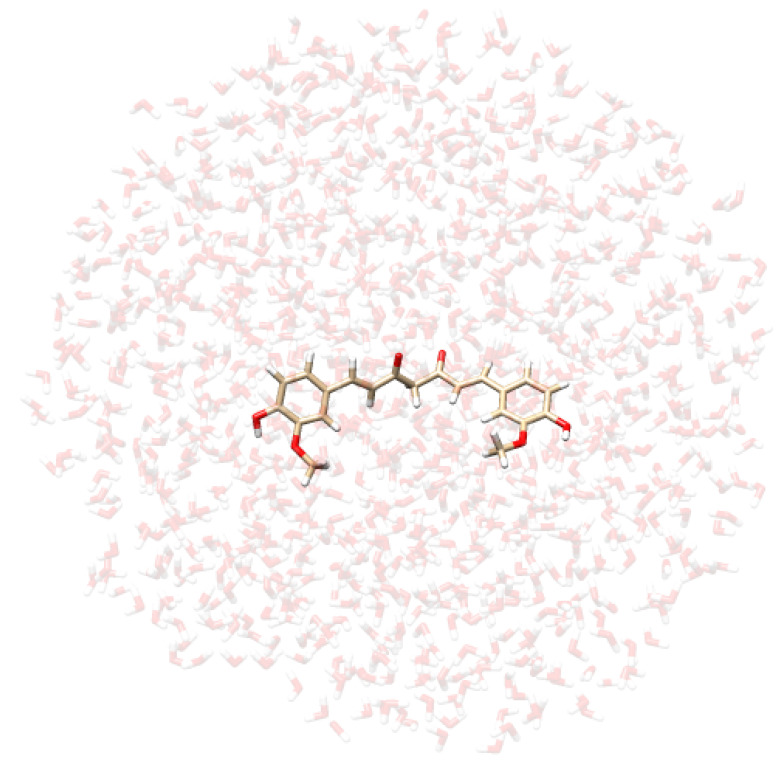	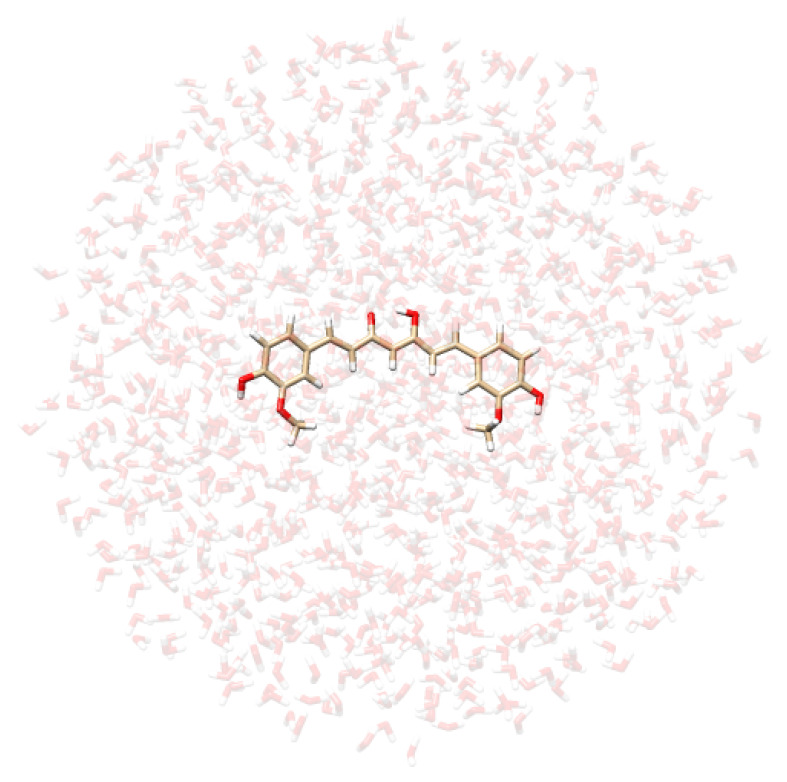

## Data Availability

The data that support the findings of this study are available from the corresponding author upon reasonable request.

## Supplementary Materials

The following materials are available online at https://www.mdpi.com/article/10.3390/molecules29184378/s1, Figures S1–S7 and Table S1: Additional MD Analysis for CYD in water and ethanol as computed using GAFF and OPLS-AA MDs; Figures S8–S15 and Tables S2 and S3: Additional spectra data computed for CYD in water and ethanol; Figures S16–S20 and Table S4: Computed data for EK and KK tautomers in aqueous solution. References [120,121,122] are cited in the Supplementary Materials.
