# Supplementary material for: Modeling UV/Vis Absorption Spectra of Food Colorants in Solution: Anthocyanins and Curcumin as Case Studies
Source: Molecules. 2024 Sep 14;29(18):4378. doi: 10.3390/molecules29184378 (PMC11434053; doi:10.3390/molecules29184378)
Supplement: Supplementary file 1 [file molecules-29-04378-s001.zip › molecules-3193615-supplementary.pdf]

Supplementary Material: Modeling Absorption Spectra of Food Colorants in Aqueous Solution: Anthocyanins and Curcumin as Case Studies

Sara Gómez,[†] Piero Lafiosca,[†] and Tommaso Giovannini[*,‡]

†*Scuola Normale Superiore, Piazza dei Cavalieri 7, 56126 Pisa, Italy.*

‡*Department of Physics, University of Rome Tor Vergata, Via della Ricerca Scientifica 1, 00133, Rome, Italy*

E-mail: tommaso.giovannini@uniroma2.it

S1 Cyanidin (CYD) Dissolved in Water and Ethanol

S1.1 MD Analysis and Hydrogen Bonding Patterns

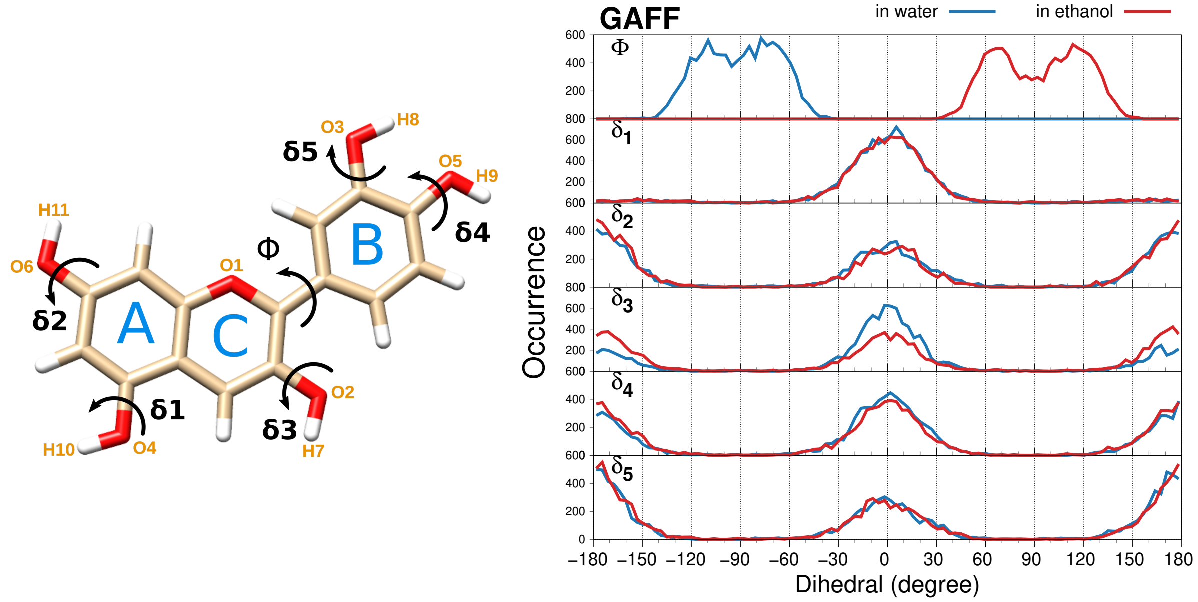

Figure S1: (left) CYD molecular structure of labeling adopted in this work. (right) Dihedral Distribution Functions (DDF) of the flexible torsions of CYD solvated in water and ethanol as calculated from GAFF MD.

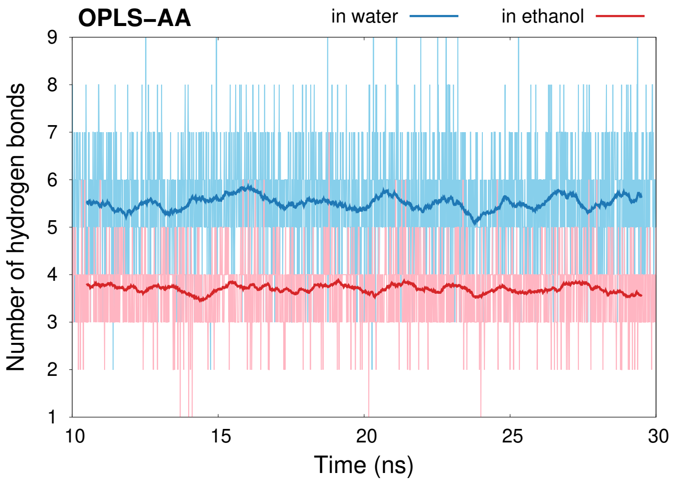

Figure S2: Evolution in time of the number of hydrogen bonds between CYD and its surrounding solvent molecules. FF: OPLS-AA

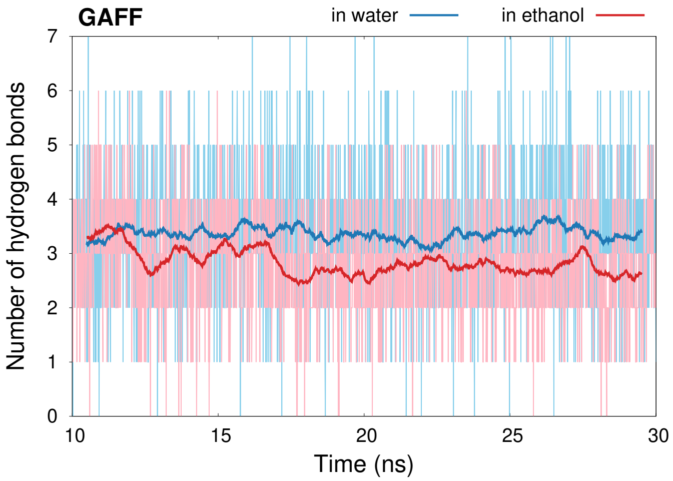

Figure S3: Evolution in time of the number of hydrogen bonds between cyanidin and its surrounding solvent molecules. FF: GAFF

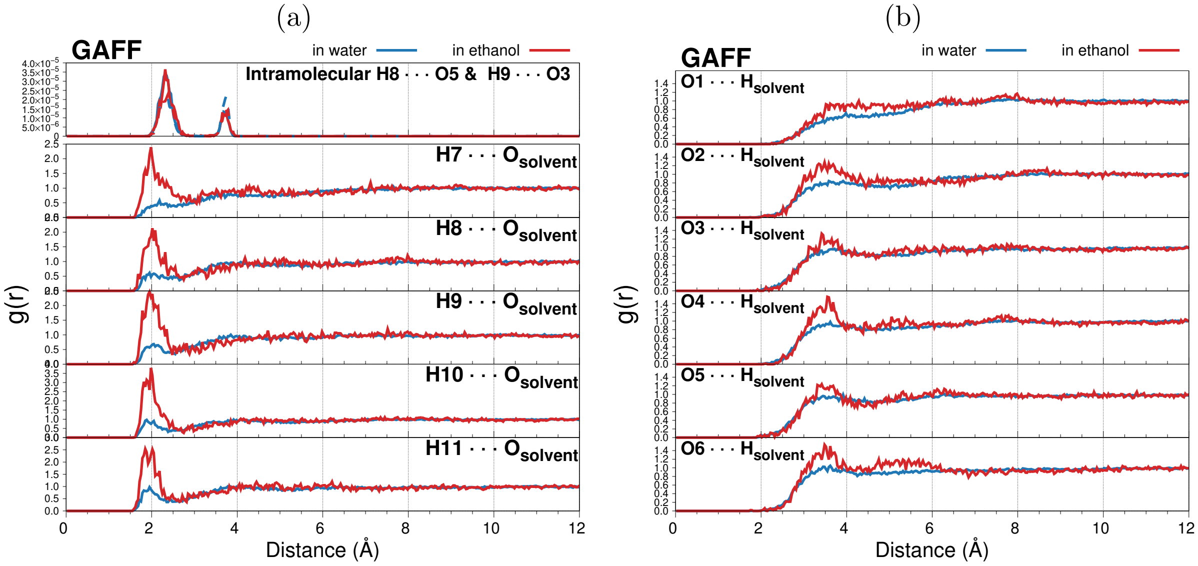

Figure S4: Radial distribution functions g(r) of intramolecular (top) and intermolecular interactions between selected H_i atoms of CYD and the solvent oxygen atoms ($H_i \cdots O_{solvent}$, left panel) and between the O_i atoms of CYD and the solvent hydrogen atoms ($O_i \cdots H_{solvent}$, right panel). All RDFs are computed along the GAFF MD trajectories.

Table S1: Position of the first peak in the RDFs and running coordination number (RCN) between the hydroxyl hydrogens in CYD (H_i, i = 1–6) and the solvent oxygen atoms ($O_{solvent}$, see also Figure S1). The data are extracted from OPLS-AA (left) and GAFF (right) MD runs. - means that no peaks are detected. The average number of hydrogen bonds $< n_{HB} >$ is also reported.

	OPLS-AA				GAFF			
	Water		Ethanol		Water		Ethanol	
	max at (Å)	RCN	max at (Å)	RCN	max at (Å)	RCN	max at (Å)	RCN
$H7 \cdots O_{solvent}$	1.82	0.99	1.82	0.87	–	–	1.98	0.97
$H8 \cdots O_{solvent}$	1.85	0.87	1.88	0.94	1.95	0.90	2.02	0.66
$H9 \cdots O_{solvent}$	1.82	1.02	1.88	0.94	2.05	0.80	1.92	1.00
$H10 \cdots O_{solvent}$	1.78	0.97	1.88	0.34	1.88	0.77	1.98	0.91
$H11 \cdots O_{solvent}$	1.78	1.02	1.82	0.86	1.95	0.75	1.88	0.64
$< n_{HB} >$	5.5		3.7		3.4		2.8	

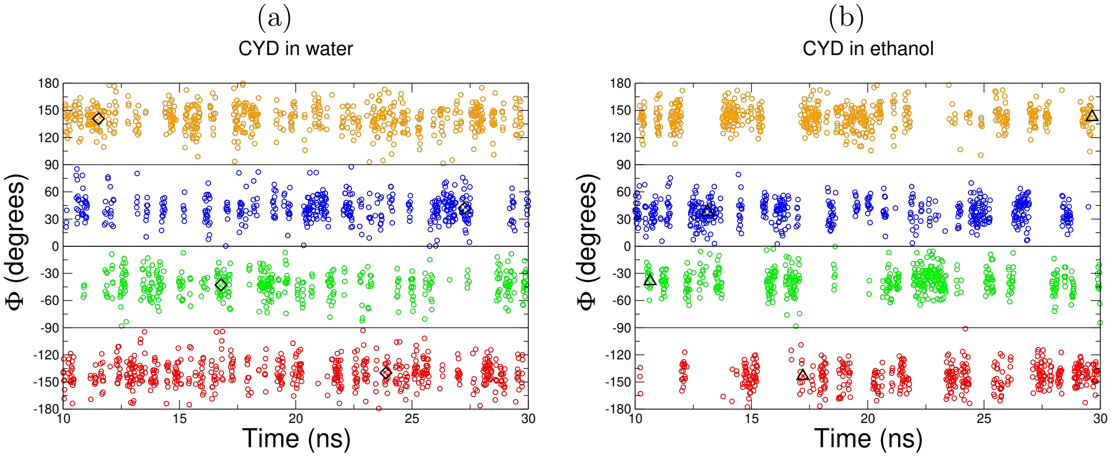

Figure S5: Time evolution of the CYD Φ dihedral angle and definition of the boundaries to clusterized CYD structures along the OPLS-AA MD trajectories. Conformers are grouped by colors and the central structure of each (which is highlighted with a diamond/triangle) is considered to be the representative structure of the cluster.

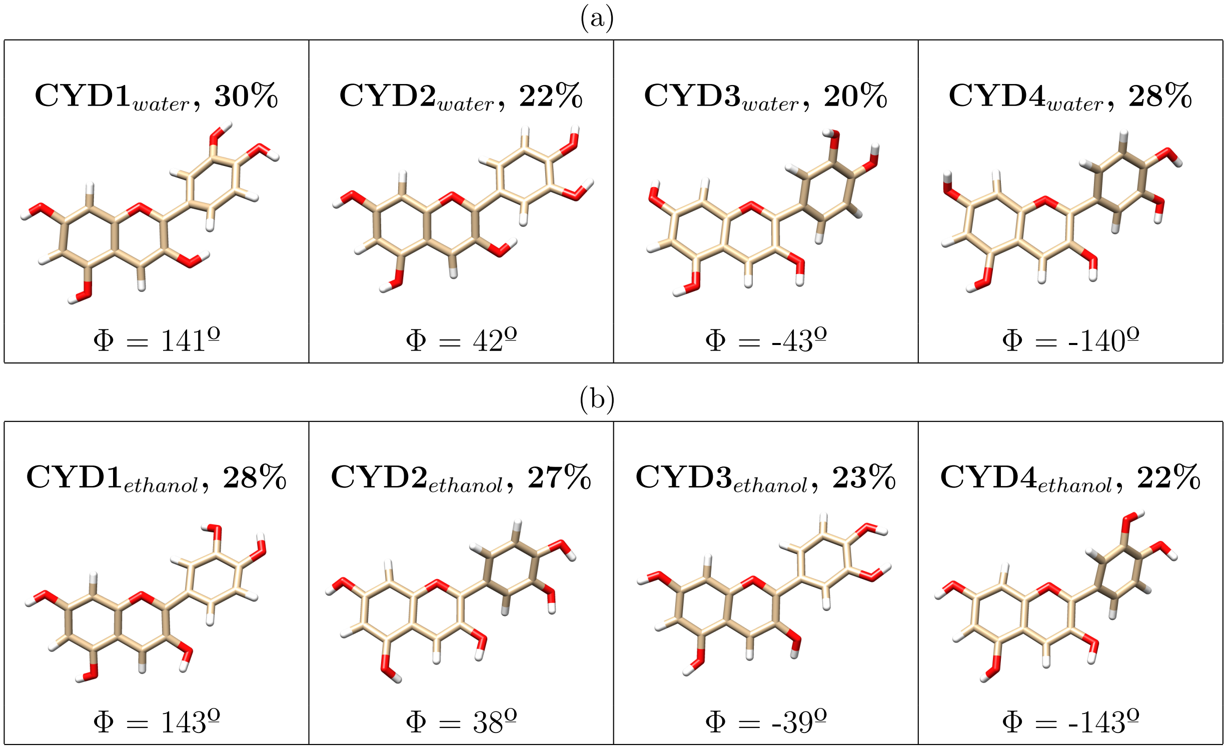

Figure S6: Dominant CYD conformers in (a) water and in (b) ethanol as resulting from the clustering analysis of the OPLS-AA MD trajectories.

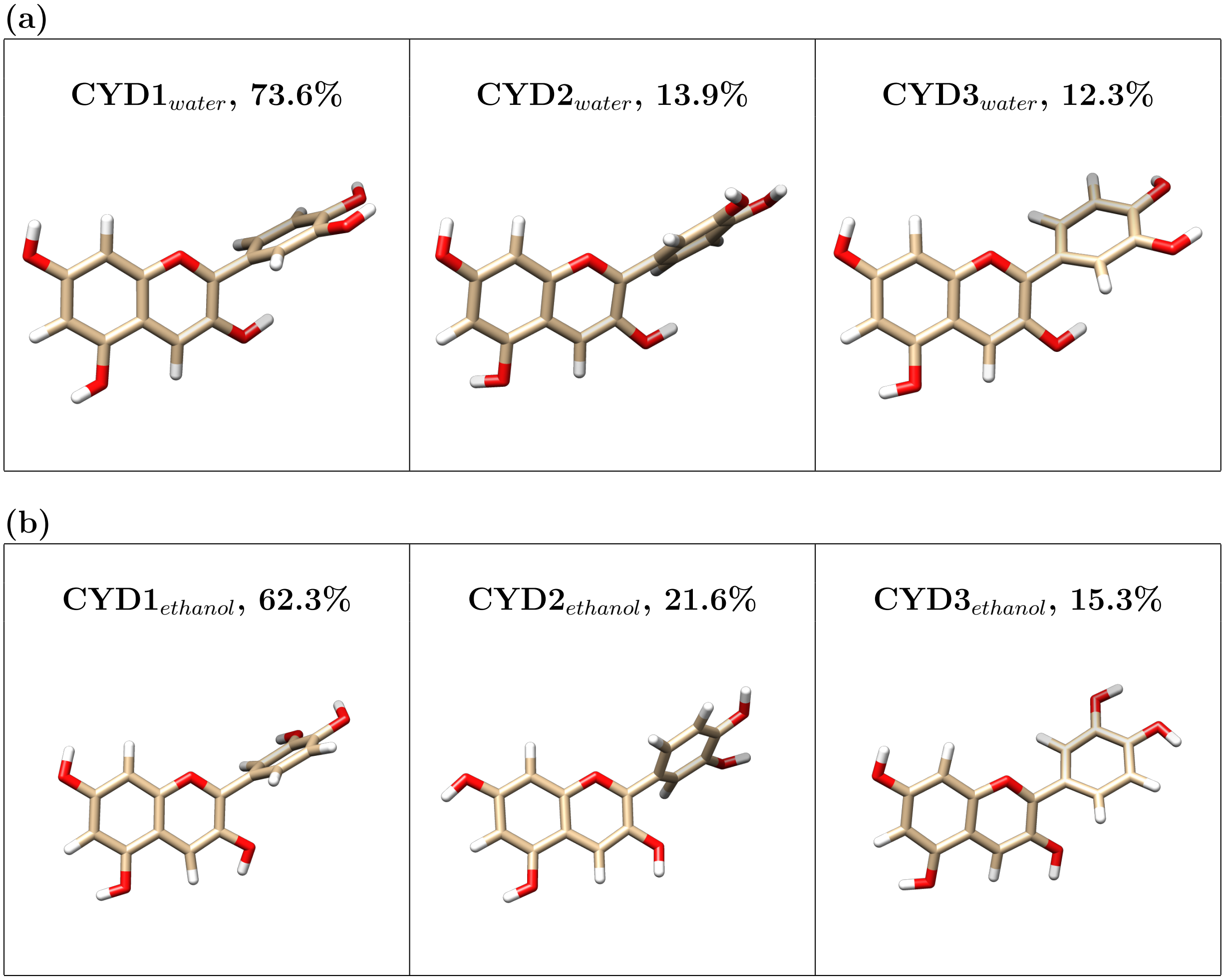

Figure S7: Dominant CYD conformers in (a) water and in (b) ethanol as resulting from the clustering analysis of the GAFF MD trajectories. Cutoff: 0.04 nm.

S1.2 Absorption Spectra: additional spectral data

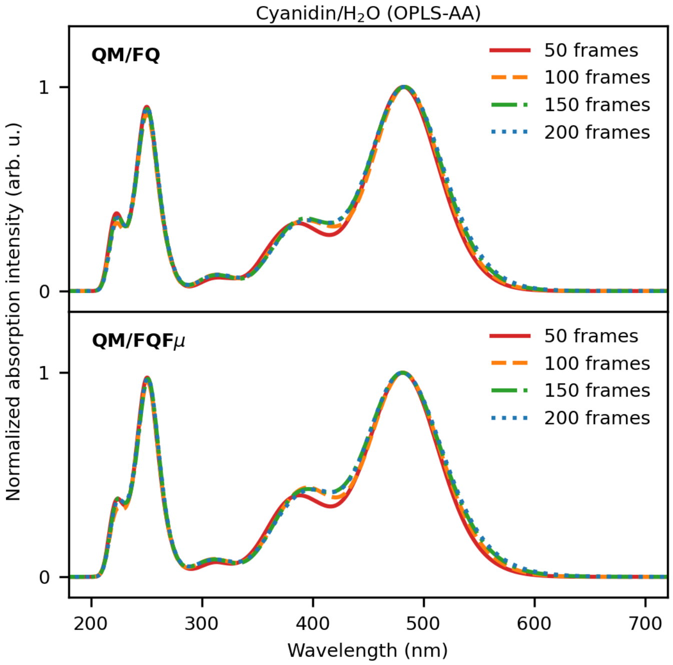

Figure S8: Computed absorption spectra of CYD in aqueous solution as a function of the number of frames extracted from OPLS-AA MD.

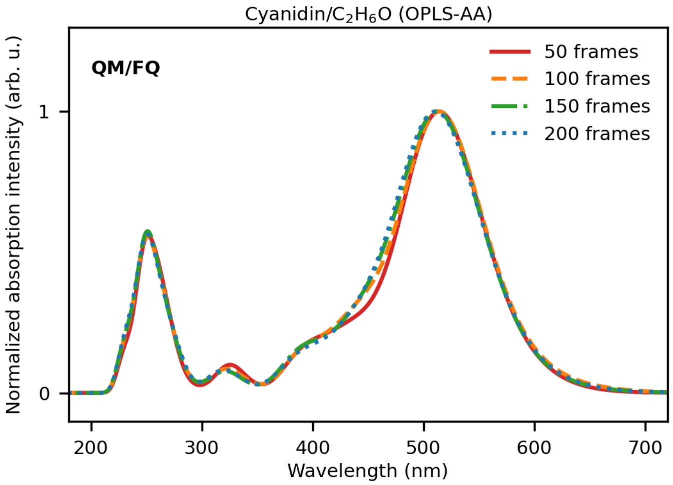

Figure S9: Computed absorption spectra of CYD in ethanol as a function of the number of frames extracted from OPLS-AA MD.

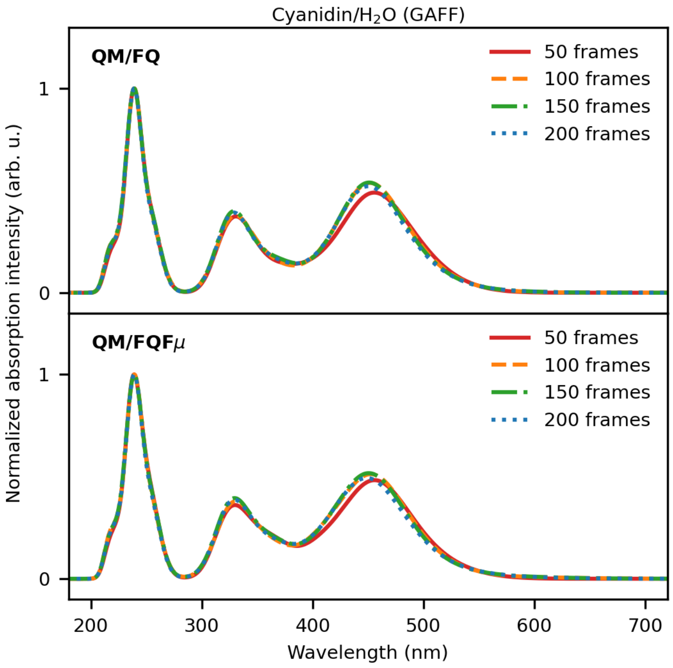

Figure S10: Computed absorption spectra of CYD in aqueous solution as a function of the number of frames extracted from GAFF MD.

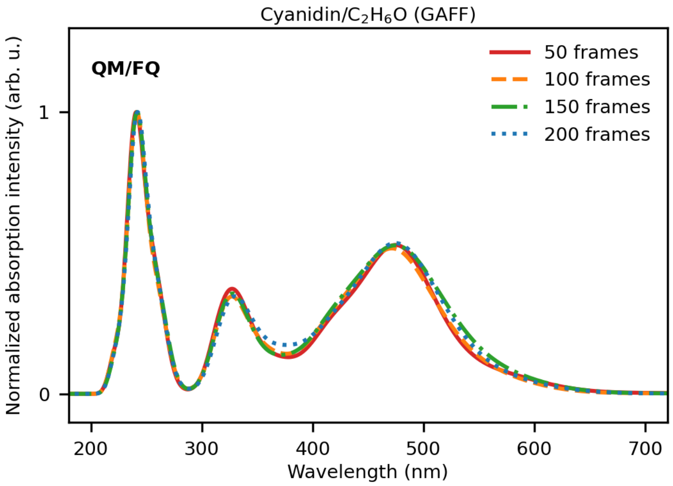

Figure S11: Computed absorption spectra of CYD in ethanol as a function of the number of frames extracted from GAFF MD.

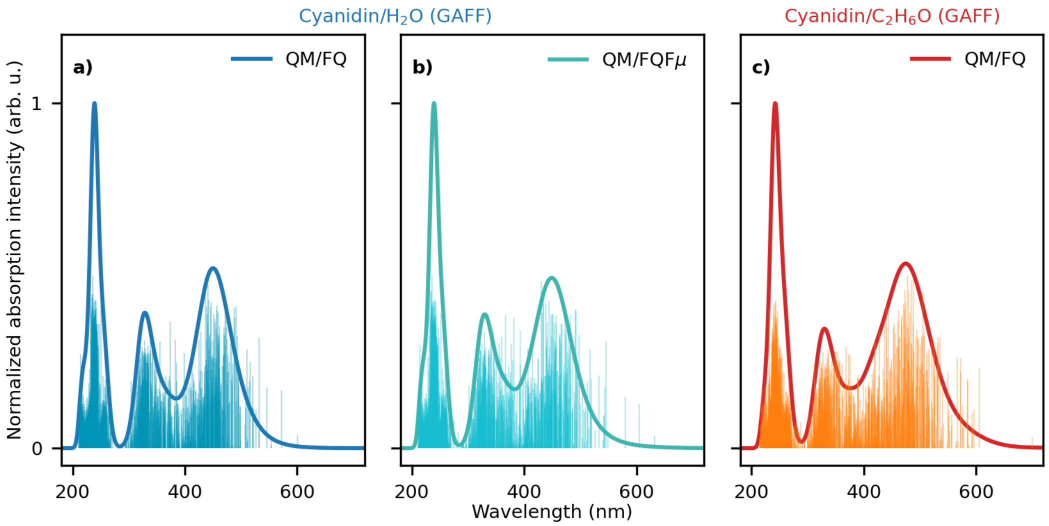

Figure S12: Stick and convoluted (normalized) spectra of solvated CYD in a) water at the QM/FQ level; b) water at the QM/FQFμ level; and c) ethanol at the QM/FQ level. All spectra are computed using 200 frames extracted from GAFF MD runs.

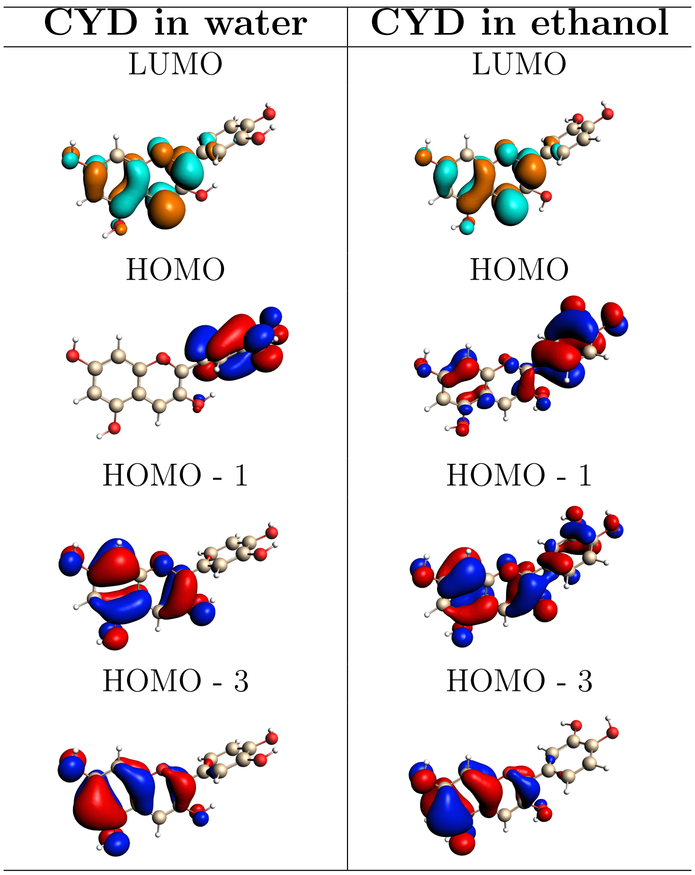

Figure S13: QM/FQ MOs involved in the vertical transitions of CYD in water (left) and in ethanol (right) computed for a representative structure extracted from GAFF MD runs.

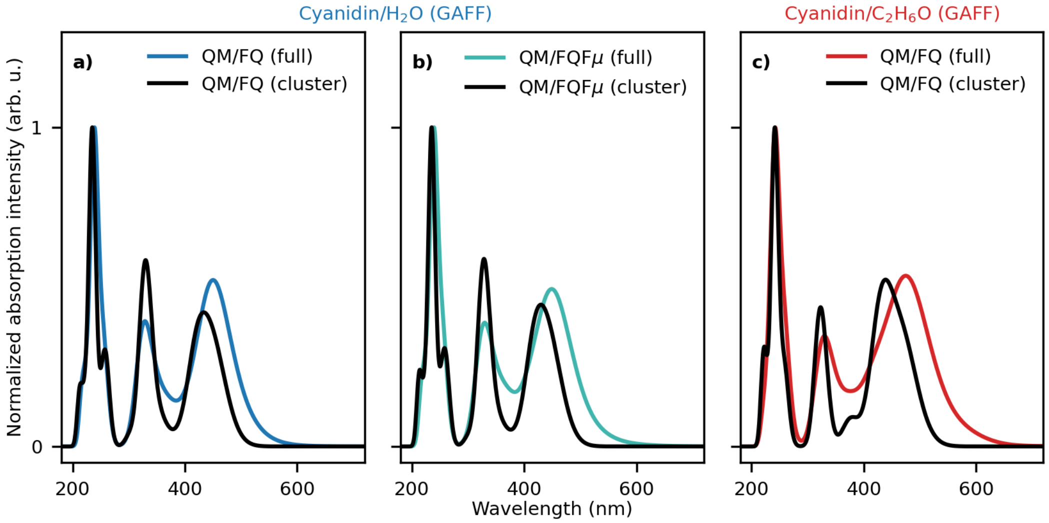

Figure S14: UV-Vis spectra of solvated CYD computed using all frames ("full") and the representative structures resulting from clustering ("cluster") of the GAFF MD runs.

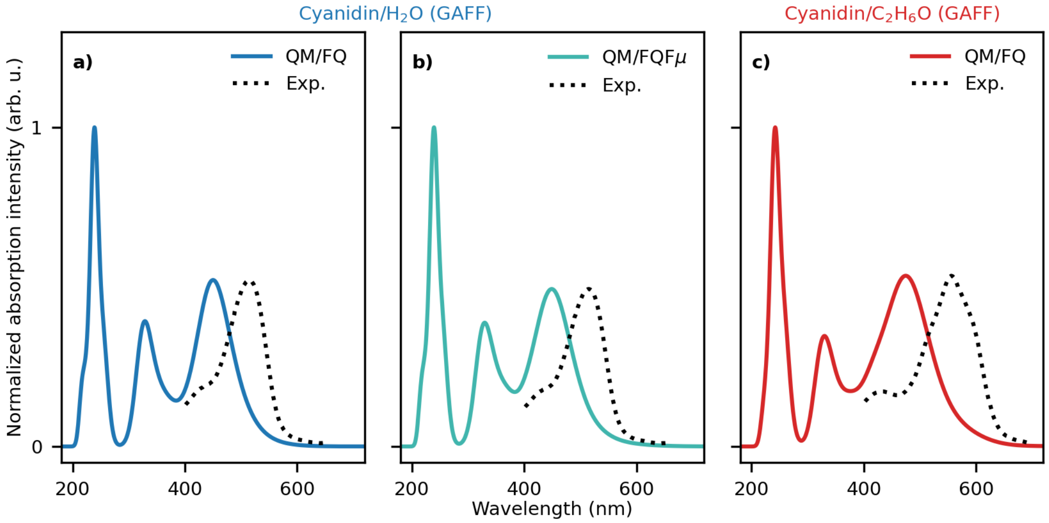

Figure S15: Computed and experimental UV-Vis spectra of solvated CYD. QM/MM frames are extracted from GAFF MDs. Experimental data are taken from Ref. 1.

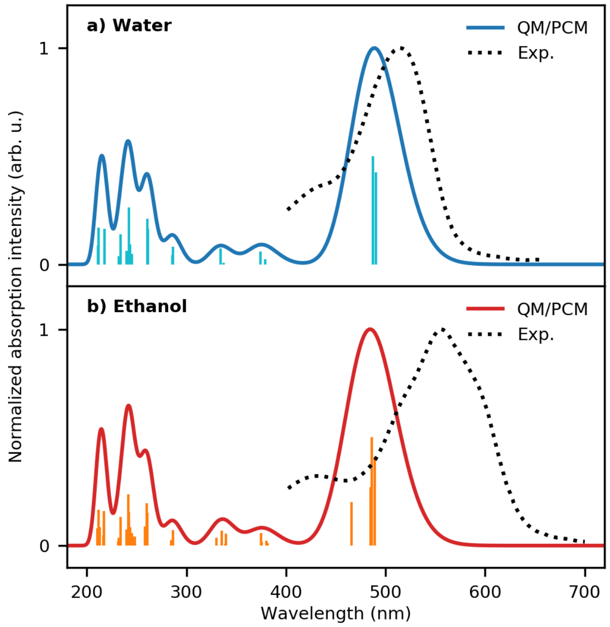

Figure S16: UV/Vis spectra of CYD in water (top) and ethanol (bottom) as computed using the implicit QM/COSMO.[2] The geometries of each initial structure (obtained by following the procedure reported in the main text) are optimized at the CAMY-B3LYP/TZP/PCM level of theory. The final number of relevant CYD conformers is 2 in water and 4 in ethanol. The relative populations obtained from the Gibbs free energy including thermal effects at 298.15 K are (0.531 and 0.469 – CYD in water) and (0.200, 0.166, 0.342, and 0.292 – CYD in ethanol). The final spectra (solid line) are obtained by convoluting the stick spectra (10 states) by using a Gaussian-type function (FWHM=0.3 eV). As a reference, experimental data taken from Ref. 1 are also reported (dotted line).

Table S2: Vertical excitation energies (VEE) of solvated CYD in water and ethanol as computed based on GAFF and OPLS-AA MD trajectories. Experimental data are taken from Ref. 1.

FF	System	Exp.		QM/FQ		QM/FQFμ	
		λ_{max} (nm)	VEE (eV)	λ_{max} (nm)	VEE (eV)	λ_{max} (nm)	VEE (eV)
GAFF	Cyanidin/H_2O	513	2.42	450	2.75	448	2.77
	Cyanidin/C_2H_6O	558	2.22	475	2.61	-	-
OPLS	Cyanidin/H_2O	513	2.42	483	2.57	481	2.58
	Cyanidin/C_2H_6O	558	2.22	512	2.42	-	-

Table S3: Survey of several works in the literature where the maximum in the electronic absorption spectrum of CYD and cyanidin has been measured (Exp) or computed (Comp). Note that, since the sugar moiety is not optically active, the absorption spectra of cyanin and cyanidin are expected to be very similar.[3]

Author	Species	Solvent	Exp	Comp
Dai and Rabani[1]	cyanidin	water, pH 1	513	
		ethanol	558	
Mazza and Brouillard[4]	cyanidin 3,5-diglucoside	water, pH 2.7	509.2	
Harborne[5]	cyanidin	ethanol	545	
Ferreira da Silva et al.[6]	cyanidin-3,5-diglucoside	water	508	
Rustioni et al.[7]	cyanidin-3-glucoside	polar	516	
Rusishvili et al.[8]	cyanidin-3-O-glucoside	water, pH 1	510	505
Calzolari et al.[3]	cyanidin-3-glucoside	water, pH 4	512	579
Ekanayake et al.[9]	cyanidin	ethanol, PCM	532	494
Barraza-Jiménez et al.[10]	cyanidin	water, PCM		491.2
		ethanol, PCM		490.4
Timrov et al.[11]	cyanidin-3-glucoside (cyanin)	water		520
Cacelli et al.[12]	cyanidin	ethanol, explicit		529.8
Barone et al.[13]	cyanidin	water, PCM		518
Malcıoğlu et al.[14]	cyanidin-3-glucoside	water, explicit		621
Soto-Rojo et al.[15]	cyanidin-3-glucoside	PCM, water		511

S2 Curcumin tautomers in aqueous solution

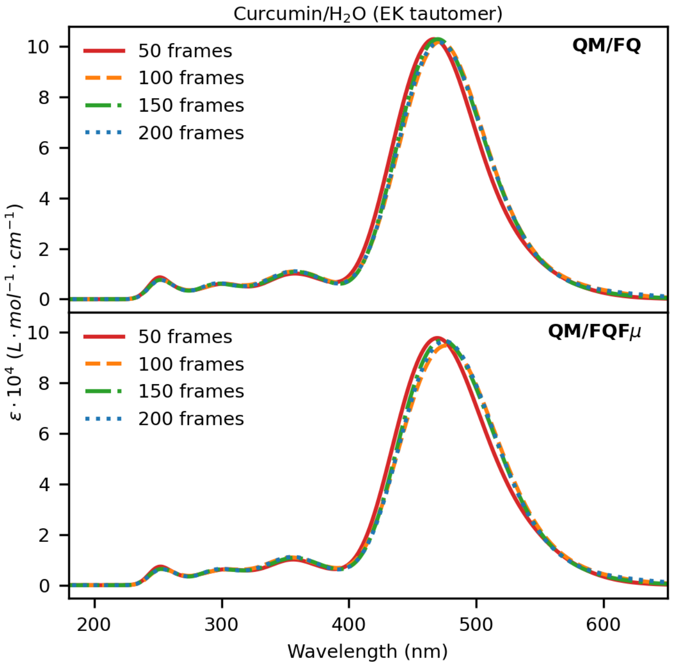

Figure S17: Computed absorption spectra of Curcumin EK tautomer in aqueous solution as a function of the number of frames.

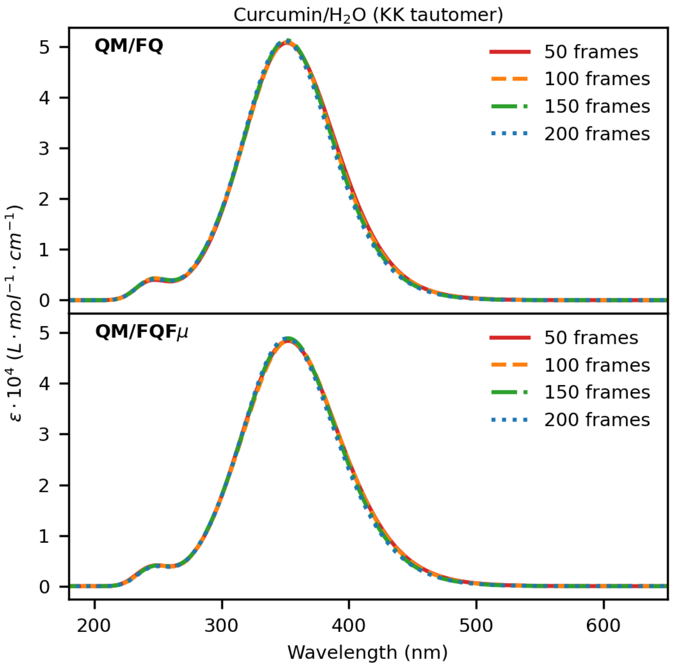

Figure S18: Computed absorption spectra of Curcumin KK tautomer in aqueous solution as a function of the number of frames.

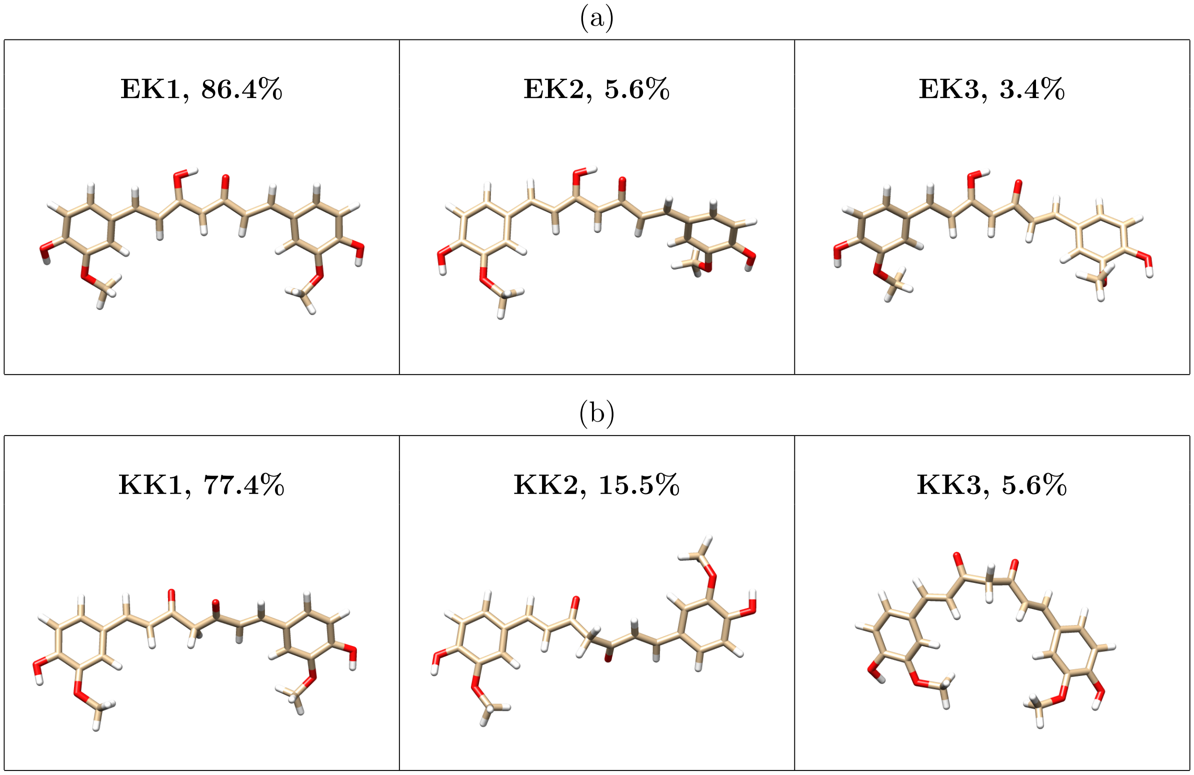

Figure S19: Dominant (>0.5%) EK and KK conformers in aqueous solution resulting from the clustering analysis of the MD trajectory. Cutoff: 0.09 nm.

Table S4: Experimental[16] and calculated maximum wavelengths (λ_{max} in nm), molar absorptivities (ε in L cm^{1} mol^{1}) for the main transition of the EK and KK tautomers in aqueous solution.

System	Exp.		QM/FQ		QM/FQFμ	
	λ_{max}	ε	λ_{max}	ε	λ_{max}	ε
EK	429	82022	471	101605	475	96472
KK	340	27993	350	51096	351	48848

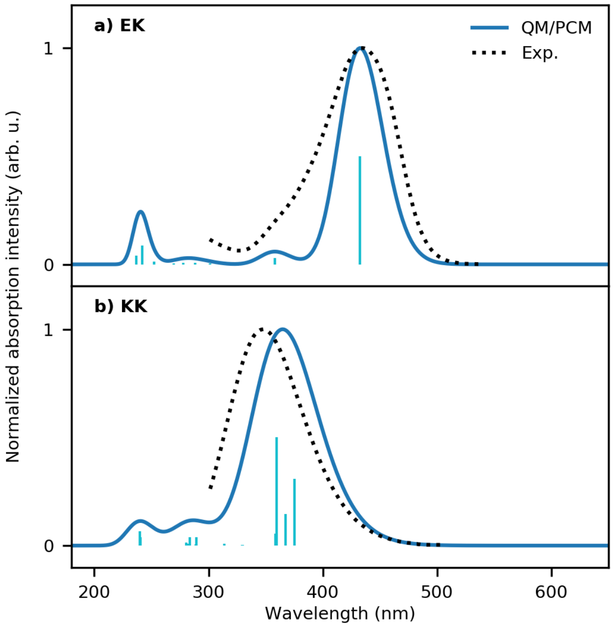

Figure S20: UV/Vis spectra of EK (top) and KK (bottom) tautomers in aqueous solution as computed by using the implicit QM/COSMO.[2] The geometries of each initial structure are taken from Ref. 16, and are optimized at the CAMY-B3LYP/TZP/PCM level of theory. In particular, a single main conformer is used for the EK tautomer and three main conformers for the KK tautomer (KK1, KK2, KK3). The KK populations obtained from the Gibbs free energy including thermal effects at 298.15 K are (< 0.001, 0.204, 0.796). The final spectra (solid line) are obtained by convoluting the stick spectra (10 states) by using a Gaussian-type function (EK: FWHM=0.3 eV; KK: FWHM=0.6 eV). As a reference, experimental data taken from Ref. 16 are also reported (dotted line).

References

(1) Dai, Q.; Rabani, J. Photosensitization of nanocrystalline TiO2 films by anthocyanin dyes. *J. Photochem. Photobiol. A* **2002**, *148*, 17–24.

(2) Klamt, A.; Schüürmann, G. COSMO: a new approach to dielectric screening in solvents with explicit expressions for the screening energy and its gradient. *Journal of the Chemical Society, Perkin Transactions 2* **1993**, 799–805.

(3) Calzolari, A.; Varsano, D.; Ruini, A.; Catellani, A.; Tel-Vered, R.; Yildiz, H.; Ovits, O.; Willner, I. Optoelectronic properties of natural cyanin dyes. *J. Phys. Chem. A* **2009**, *113*, 8801–8810.

(4) Mazza, G.; Brouillard, R. The mechanism of co-pigmentation of anthocyanins in aqueous solutions. *Phytochemistry* **1990**, *29*, 1097–1102.

(5) Harborne, J. Spectral methods of characterizing anthocyanins. *Biochemical Journal* **1958**, *70*, 22.

(6) Ferreira da Silva, P.; Paulo, L.; Barbafina, A.; Elisei, F.; Quina, F. H.; Maçanita, A. L. Photoprotection and the photophysics of acylated anthocyanins. *Chemistry–A European Journal* **2012**, *18*, 3736–3744.

(7) Rustioni, L.; Di Meo, F.; Guillaume, M.; Failla, O.; Trouillas, P. Tuning color variation in grape anthocyanins at the molecular scale. *Food Chem.* **2013**, *141*, 4349–4357.

(8) Rusishvili, M.; Grisanti, L.; Laporte, S.; Micciarelli, M.; Rosa, M.; Robbins, R. J.; Collins, T.; Magistrato, A.; Baroni, S. Unraveling the molecular mechanisms of color expression in anthocyanins. *Phys. Chem. Chem. Phys.* **2019**, *21*, 8757–8766.

(9) Ekanayake, P.; Kooh, M. R. R.; Kumara, N.; Lim, A.; Petra, M. I.; Voo, N. Y.; Lim, C. M. Combined experimental and DFT–TDDFT study of photo-active con-

stituents of Canarium odontophyllum for DSSC application. *Chem. Phys. Lett.* **2013**, *585*, 121–127.

(10) Barraza-Jiménez, D.; Flores-Hidalgo, H. I.; Torres-Herrera, S. I.; Olvera-Corral, R. A.; Flores-Hidalgo, M. A. In *Photocatalysts*; Awwad, N. S., Alarfaji, S. S., Alomary, A., Eds.; IntechOpen: Rijeka, 2022; Chapter 11.

(11) Timrov, I.; Micciarelli, M.; Rosa, M.; Calzolari, A.; Baroni, S. Multimodel approach to the optical properties of molecular dyes in solution. *J. Chem. Theory Comput.* **2016**, *12*, 4423–4429.

(12) Cacelli, I.; Ferretti, A.; Prampolini, G. Predicting light absorption properties of anthocyanidins in solution: a multi-level computational approach. *Theor. Chem. Acc.* **2016**, *135*, 1–17.

(13) Barone, V.; Ferretti, A.; Pino, I. Absorption spectra of natural pigments as sensitizers in solar cells by TD-DFT and MRPT2: protonated cyanidin. *Phys. Chem. Chem. Phys.* **2012**, *14*, 16130–16137.

(14) Malcıoğlu, O. B.; Calzolari, A.; Gebauer, R.; Varsano, D.; Baroni, S. Dielectric and thermal effects on the optical properties of natural dyes: A case study on solvated cyanin. *J. Am. Chem. Soc.* **2011**, *133*, 15425–15433.

(15) Soto-Rojo, R.; Baldenebro-López, J.; Flores-Holguín, N.; Glossman-Mitnik, D. Comparison of several protocols for the computational prediction of the maximum absorption wavelength of chrysanthemin. *J. Mol. Model.* **2014**, *20*, 1–9.

(16) Puglisi, A.; Giovannini, T.; Antonov, L.; Cappelli, C. Interplay between conformational and solvent effects in UV-visible absorption spectra: Curcumin tautomers as a case study. *Phys. Chem. Chem. Phys.* **2019**, *21*, 15504–15514.
